# Statistical tests for detecting variance effects in quantitative trait studies

**DOI:** 10.1093/bioinformatics/bty565

**Published:** 2018-07-06

**Authors:** Bianca Dumitrascu, Gregory Darnell, Julien Ayroles, Barbara E Engelhardt

**Affiliations:** 1 Lewis-Sigler Institute for Integrative Genomics, Princeton University, Princeton, NJ, USA; 2 Department of Ecology and Evolutionary Biology, Princeton University, Princeton, NJ, USA; 3 Department of Computer Science, Princeton University, Princeton, NJ, USA; 4 Center for Statistics and Machine Learning, Princeton University, Princeton, NJ, USA

## Abstract

**Motivation:**

Identifying variants, both discrete and continuous, that are associated with quantitative traits, or QTs, is the primary focus of quantitative genetics. Most current methods are limited to identifying mean effects, or associations between genotype or covariates and the mean value of a quantitative trait. It is possible, however, that a variant may affect the variance of the quantitative trait in lieu of, or in addition to, affecting the trait mean. Here, we develop a general methodology to identify covariates with variance effects on a quantitative trait using a Bayesian heteroskedastic linear regression model (BTH). We compare BTH with existing methods to detect variance effects across a large range of simulations drawn from scenarios common to the analysis of quantitative traits.

**Results:**

We find that BTH and a double generalized linear model (dglm) outperform classical tests used for detecting variance effects in recent genomic studies. We show BTH and dglm are less likely to generate spurious discoveries through simulations and application to identifying methylation variance QTs and expression variance QTs. We identify four variance effects of sex in the Cardiovascular and Pharmacogenetics study. Our work is the first to offer a comprehensive view of variance identifying methodology. We identify shortcomings in previously used methodology and provide a more conservative and robust alternative. We extend variance effect analysis to a wide array of covariates that enables a new statistical dimension in the study of sex and age specific quantitative trait effects.

**Availability and implementation:**

https://github.com/b2du/bth.

**Supplementary information:**

Supplementary data are available at *Bioinformatics* online.

## 1 Introduction

Identifying covariates in a population that are associated with complex quantitative traits (QTs) is central to the study of statistical genetics (Stranger *et al.*, 2007; Zeggini *et al.*, 2007). Quantitative trait loci (QTLs) are genetic variants that are associated with differences in mean phenotype values within a population. Recently, variance QTLs (vQTLs), or genetic variants associated with differences in the variance of a quantitative trait, have been observed in genetic studies (Ayroles *et al.*, 2015; Brown *et al.*, 2014; Metzger *et al.*, 2015; Paré *et al.*, 2010; Yang *et al.*, 2012). These studies include diverse quantitative phenotypes, including left-right turning tendency in the fruit fly *Drosophila melanogaster* (Ayroles *et al.*, 2015), coat color in the rock pocket mice *Chaetodipus intermedius* (Nachman *et al.*, 2003), and thermotolerance (Queitsch *et al.*, 2002) and flowering time (Salomé *et al.*, 2011) in the plant *Arabidopsis thaliana*.

These variance-associated covariates have wide ranging implications in phenotypic variance. Phenotypic variability offers an adaptive evolutionary solution to changing environments (Gibson and Wagner, 2000; Queitsch *et al.*, 2002), and indicates the presence of other complex effects such as epistasis (Brown *et al.*, 2014; Paré *et al.*, 2010) or canalization (Gibson and Wagner, 2000). In medical genetics, where disease states often emerge beyond a phenotypic threshold (Wang *et al.*, 2017), controlling phenotypic variability allows control over the proportion of individuals that exceed that threshold while population means are preserved (Ayroles *et al.*, 2015). Robust statistical methods to identify variance effects are therefore essential to characterize the role that population covariates with variance effects on quantitative traits, or vQTCs, play in the regulation of complex traits, including disease risk.

**Fig. 1.**
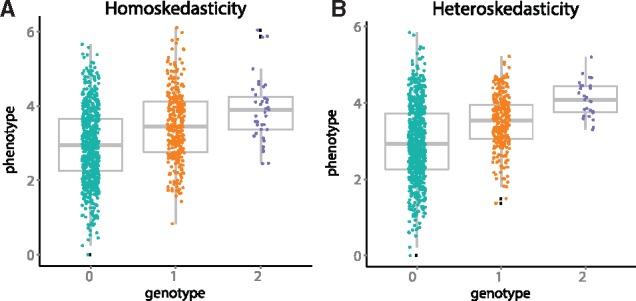
Example of heteroskedasticity for biallelic variation. The x-axis is genotypes represented as the number of copies of the minor allele. The y-axis is the quantitative trait values across individuals sampled from a population. Panel **A**: *Homoskedasticity*, where each trait distribution from three genotypes have equal variance. Panel **B**: *Heteroskedasticity*, where each trait distribution from three genotypes have different variances. The data were simulated with *n *=* *1000 with minor allele frequency *π_maf_* = 0.2; each genotype group was plotted with x-axis jitter to show data density

Methodologically, detecting vQTCs is performed using statistical tests for *heteroskedasticity*. Heteroskedasticity refers to the circumstance in which the variance of a response variable—here, a quantitative trait—is unequal across the range of values of a covariate such as genotype or age (Fig. 1). In the case of vQTCs, the quantitative traits can be gene expression levels, methylation levels, or hip-to-waist ratio. Here, we develop and validate a robust statistical test for variance effects. More broadly, we extend this approach to account for both continuous and discrete non-genetic covariates such as sex, age and BMI. While some of these covariates, such as sex and genotype, will by definition have a causal relationship with the QT, others such as BMI may not have a causal effect on the variance of a QT despite their correlation; while we use the language of ‘variance effects’ throughout, this does not imply causation for non-causal covariates.

Three methods widely used in the genetics literature (Ayroles *et al.*, 2015; Brown *et al.*, 2014; Metzger *et al.*, 2015; Paré *et al.*, 2010; Yang *et al.*, 2012) to identify vQTCs are the Levene and Brown-Forysthe tests (Brown and Forsythe, 1974; Schultz, 1985), and the correlation least squares (CLS) test (Brown *et al.*, 2014). The Levene and Brown-Forsythe tests for heteroskedasticity across *k* groups come from a similar family of ANOVA-based statistics, where the within-group variance is compared to the across-group variance. The null hypothesis for these tests is that all groups have the same variance. The two tests differ in that the Levene test uses mean statistics to compute variance whereas Brown-Forsythe uses median statistics to compute variance. The Bartlett test (Bartlett, 1937) has also been used in genomic contexts (Yang *et al.*, 2012). The Bartlett test relies on the computation of *pooled variances*, or weighted average of the groups, which are used to approximate an F-distribution, and assumes the groups come from normal distributions. While similar to the Levene test, the Bartlett test is more sensitive to departures from normality, which makes it less useful for genomic analyses (Supplementary Methods).

The CLS test first fits a linear regression model to the trait and the covariate, then tests for a correlation between the covariate and the squared residual errors of the fitted linear model using Spearman rank correlation (Brown *et al.*, 2014). The test statistic is the corresponding Spearman’s rank correlation coefficient. Related two stage tests include likelihood ratio-based tests such as *dglm* (Dunn and Smyth, 2012; Rönnegård and Valdar, 2011) or *famLRTV* (Cao *et al.*, 2015). As *famLRTV* performs similarly to the Levene test (Cao *et al.*, 2014), we did not include it in our analysis.

While the Levene, Brown-Forsythe and CLS tests are standard in various research areas, they each have drawbacks when applied to genomic data. The Levene and Brown-Forsythe tests both require categorical covariates, preventing the use of continuous covariates such as imputed genetic variants, age or methylation levels. These methods sacrifice statistical power by avoiding assumptions about the functional form of the heteroskedastic effects, allowing the variance across the covariate-defined groups to change in a non-monotone way. CLS addresses both of these drawbacks by using a standard linear model; however, because the test is performed in two stages—neither of which incorporate uncertainty explicitly—CLS is prone to overfitting.

Less common in genomics research, *dglm* is a parametric approach that models variance explicitly and cycles over two stages until convergence (Verbyla and Smyth, 1998). First, it fits a linear predictor of the variance by taking the estimated squared residuals from the fit of a weighted linear model. Second, it uses the fit of a generalized linear model (GLM) with variance as its response to update the weighted linear model of the first step. This approach is effective because it uses a GLM framework to capture the possible heteroskedasticity in the data; as with CLS, it does not incorporate uncertainty in the point estimate from the first stage. However, the main drawback of *dglm* is its numerical instability, which makes it difficult to apply to large genomic data. This numerical instability often arises in the context of low minor allele frequency, which makes the method challenging to apply from a practitioner’s point of view.

In this study, we propose a flexible Bayesian strategy for detecting genotypic loci and covariates with effects on phenotypic variance. Our method can incorporate both discrete and continuous covariates, and leads to stable, effective inference. We show through extensive simulations that it outperforms similar tests that are routinely used in genomic studies. On real data, where alternative methods generate hundreds of hits, this fact has important implications, suggesting not only that our test is robust and conservative, but that alternative tests are poorly calibrated and lead to spurious results.

## 2 Approach

### 2.1 A Bayesian test for heteroskedasticity

The Bayesian test for heteroskedasticity (BTH) models a continuous trait across *n* samples, $y\in R^{n}$, with a Gaussian distribution, where both the mean and variance parameters are functions of the covariate $x\in R^{n}$, $y_{i}∼N(\beta_{0}+\beta x_{i},\sigma^{2}\alpha^{-x_{i}})$. Here, *β*_0_ ∝ 1 is the *y*-axis intercept, $\beta ∼N(0,\mu^{-1})$ is the regression coefficient (or the *mean effect size*), $\sigma^{2}∼InvGa(\theta_{1},\theta_{2})$ is the residual variance, and *α* is the heteroskedastic effect with a prior log α∼Cauchy(0,ν). When *α *= 1, the variance of the response is not a function of the covariate, whereas when *α *≠ 1, the variance term is associated with the covariate. We put priors on each of these parameters in order to incorporate biologically appropriate and computationally tractable forms of uncertainty in the test (Supplemental Materials).

Using this model, we computed Bayes factors (Kass and Raftery, 1995) (BFs) to compare the likelihood of the data under the null hypothesis (*H*_0_, *α *= 1) with the likelihood of the data under the alternative hypothesis (*H_A_*, *α *≠ 1). In particular, for each application of the model (e.g. one covariate x and one quantitative trait y across *n* individuals), the BF has the form

(1) $$BF(y,x)=\frac{\mathrm{Pr}(y|x,(H_{A},\alpha \neq 1))}{\mathrm{Pr}(y|x,(H_{0},\alpha =1))}.$$

We compute this BF by marginalizing over the mean effect size *β* and evaluating the resulting multivariate integral using a multivariate Laplace approximation similar to the integrated nested Laplace approximation (INLA) method (Rue *et al.*, 2009; Ruiz-Cárdenas *et al.*, 2012) (Supplemental Materials).

The BFs provide a measure of the heteroskedasticity of the association between a covariate and a phenotype of interest under certain assumptions, which we examine carefully in the simulations. To quantify the global false discovery rate (FDR) of the quantified BFs, we designed and performed permutations of the covariate-trait pair such that any mean effects are maintained but variance effects are removed (Methods, below). Furthermore, we generated a distribution of *BF_perm_* corresponding to data in which the variance of the phenotype is independent of the covariate. Thus, we compute FDR by considering, for any BF threshold *t*,

(2) $$FDR(t)=\frac{\left|\{BF_{perm}|BF_{perm}>t\}\right|}{\left|\{BF|BF>t\}\right|},$$

which approximates the ratio of the number false positives versus the number of false positives and true positives across all tests for BF threshold *t*. We used the FDR-calibrated BF thresholds to discover heteroskedastic associations in our data, and we compared our discoveries to the discoveries from existing tests for heteroskedasticity.

### 2.2 Available tests of heteroskedasticity

We compared results from our BTH against four tests for heteroskedasticity: i) the Brown-Forsythe test (Brown and Forsythe, 1974); ii) the Levene test (Schultz, 1985; Shen *et al.*, 2012; Struchalin *et al.*, 2012); iii) the correlation least squares (CLS) test (Brown *et al.*, 2014); and iv) the double generalized linear model (*dglm*) test.

Each of these statistical tests makes assumptions about the underlying data by design. The Levene test, which has been used in a number of biological studies (Ayroles *et al.*, 2015; Paré *et al.*, 2010; Soave and Sun, 2017; Yang *et al.*, 2012), assumes that, in the data: i) the noise is symmetric; ii) the groups are balanced; iii) the covariate is a categorical variable; and iv) the categories are unordered, so arbitrary functions are tested. By using median statistics instead of mean statistics, the Brown-Forsythe test overcomes the assumption of symmetric noise (Brown and Forsythe, 1974). The CLS test assumes i) continuous or ordered covariates; ii) linear dosage effects of the covariate; iii) sufficient minor allele frequency (MAF). When MAF is low, as is often the case for functional variants (Nelson *et al.*, 2012), the maximum likelihood estimates from CLS will have large standard error.

Our model for BTH makes the following assumptions: i) the noise has a Gaussian distribution; ii) the covariate is a continuous or ordered value; and iii) the functional form of the heteroskedasticity is dosage or variant dependent, with monotone effects on the variance. We make these assumptions to gain statistical power in identifying heteroskedastic effects in genomic studies, and to avoid spurious results. Assumption iii) is illustrated through modelling the variance as an exponential function $\sigma^{2}\alpha^{-x_{i}}$ which is monotone with respect to the variant *x_i_*. This assumption becomes particularly meaningful in the case where the variants considered are non-binary, such as age. The *dglm* approach makes the same assumptions as BTH. In contrast to the above methods, including *dglm*, our test incorporates estimates of uncertainty, integrating over all possible mean effects in both the null and the alternative hypothesis.

We show the value of BTH with respect to these related approaches in extensive simulations and in three genomic data applications. In the simulations, for data that violate the model assumptions, we provide prescriptive tests and transformations to enable a well-powered application of BTH. We then apply BTH to methylation QTLs, gene expression QTLs and gene expression data versus biological covariates to illustrate the promise of BTH for identifying variance effects in diverse genomic data.

To compare results from BTH with state-of-the-art tests for variance QTLs, we simulated data across a range of possible scenarios in genomic studies. We account for discrete and continuous covariates, different parameter settings and a number of distributions of the quantitative trait.

### 2.3 Simulating quantitative trait data

For discrete covariates, each simulated biallelic, diploid variant *x_i_* ∈ {0, 1, 2} from individual i={1,…,n} is sampled as two independent draws from a Bernoulli distribution with bias equal to the minor allele frequency (*π_maf_*): $x_{i}∼Bin(2,\pi_{maf})$. For imputed covariates, for each individual i={1,…n}, discrete values *z_i_* ∈ {0, 1, 2} are sampled from a Bernoulli distribution: $z_{i}∼Bin(2,\pi_{maf})$. Continuous data resembling imputed genotypes are then simulated from a modified mixture of normal distributions: $x_{i}=1_{m_{i}=0}\cdot |c_{0}|+1_{m_{i}=1}\cdot c_{1}+1_{m_{i}=2}\cdot (2-|c_{2}|)$, where $c_{0},c_{2}∼N(0,0.5)$ and $c_{1}∼N(1,0.5)$, and $1_{\cdot }$ is the indicator function. This process ensures that the simulated imputed genotypes are bounded by 0 and 2, and they represent the expected value of the genotype, which is a standard representation (Howie *et al.*, 2009).

Then, given intercept *β*_0_, effect size *β*, and variance parameters *σ*^2^, *α*, we simulated the quantitative trait *y_i_* for individual *i* from a Gaussian distribution, using a linear model: $y_{i}∼N(\beta_{0}+\beta x_{i},\sigma^{2}\alpha^{-x_{i}})$. This is an ideal situation, with the heteroskedastic functional form matching that of our test. Across simulations, we sampled covariates and quantitative traits across various parameter settings: n={300,500,1000} samples, minor allele frequencies $\pi_{maf}=\{0.05,0.2,0.3\}$, mean effect size β={0,0.2,0.5,1}, the level of heteroskedasticity log α={−0.2,−0.1,0,0.1,0.2}, intercept $\beta_{0}=\{0,1\}$, and a fixed variance parameter $\sigma^{2}=1.0$. These simulations correspond well to current eQTL studies in sample size (GTEx Consortium, 2017; Battle *et al.*, 2014), minor allele frequencies (Nelson *et al.*, 2012) and effects (GTEx Consortium, 2017; Savolainen *et al.*, 2013).

For each parameter configuration, we generated 1000 simulated datasets of covariates **x** and corresponding traits **y**. For each simulation with heteroskedasticity, we performed a single permutation of the quantitative trait sample labels and included for comparison this null simulation with identical MAF and trait distribution (see Online Methods). Thus, each simulation result contains 2000 tests, half of which are from a null distribution constructed using permutations, and the other of which are simulated to have variance effects.

## 3. Results

### 3.1 Simulation results: ideal model, discrete covariates

For discrete genotypes, we compared results from BTH against results from the Brown-Forsythe test (Brown and Forsythe, 1974), the Levene test (Levene, 1961), the correlation least square test (CLS) (Brown *et al.*, 2014) and the double generalized linear model *dglm*. We compared performance using precision-recall curves, which quantify the proportion of true associations discovered (x-axis: recall or statistical power) versus the proportion of discoveries that are truly associated (y-axis: precision, or 1–FDR). When the curves are close to precision = 0.5 across most values of recall, this means that the method cannot differentiate between non-associations and true associations in this scenario with equal numbers of true and null associations. The closer the curves are to precision = 1 across values of recall, the greater the area under the curve (AUC) is (with a maximum of one), and the better the performance of that method.

**Fig. 2.**
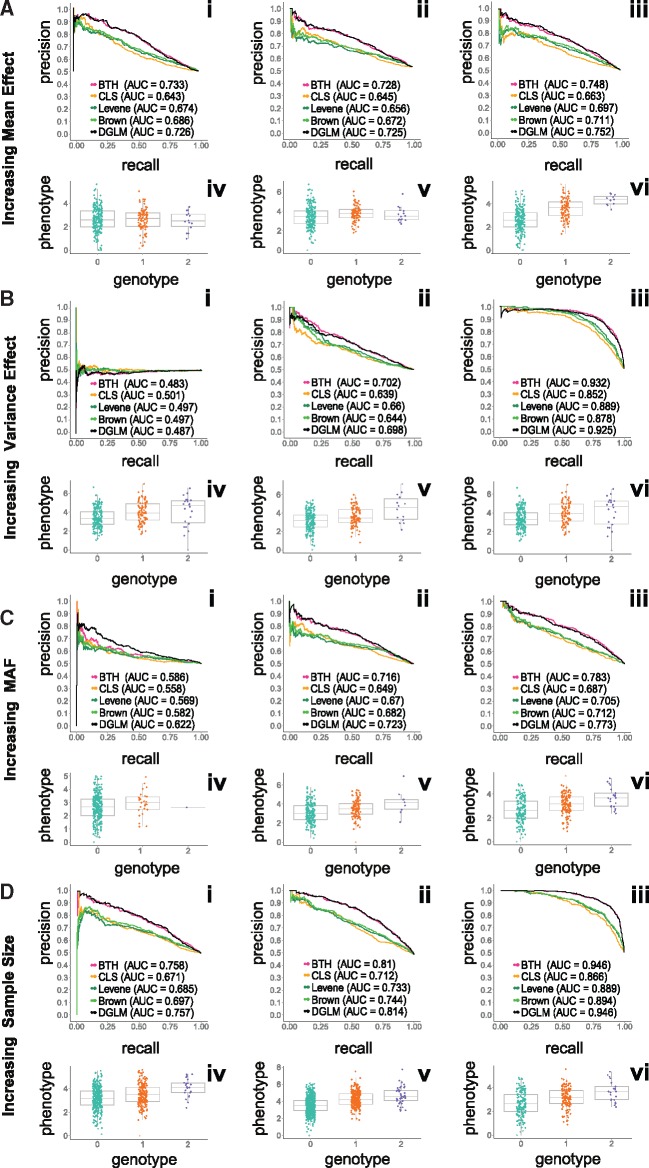
Precision-recall curves comparing performance of BTH versus three other methods and example plots of underlying discrete simulated data. Panel **A**: increasing mean effect size: *π_maf_* = 0.2, *n *=* *300, β∈{0,0.2,1},  log(α)=0.1; Panel **B**: increasing the variance effects: $\pi_{maf}=0.2$, *n *=* *300, β=0.5,  log(α)∈{0,−0.1,−0.2}; Panel **C**: increasing minor allele frequency: $\pi_{maf}\in \{0.05,0.2,0.3\}$, *n *=* *300, β=0.5,  log(α)=0.1; Panel **D**: increasing sample size: $\pi_{maf}=0.2,\;n\in \{300,500,1000\},\;\beta =0.5,\;\;\text{log}(\alpha )=0.1$

In the results of the simulations we found that, as the variance effects in the simulated data grow, it becomes easier for the tests to identify these effects (Fig. 2B—ii, iii); moreover, the permutation appears to generate a true null (Fig. 2B—i) under these ideal simulation assumptions. Here, the benefits of BTH and *dglm* are illustrated: when variance effect log (*α*) = –0.2, we see at high levels of recall as much as a 10% improvement in precision (Fig. 2B—iii). Considering mean effects, across most recall values BTH and *dglm* show consistently higher AUC than other methods (Fig. 2A). For BTH, this trend illustrates the fact that a Gaussian prior on the mean effect is robust as mean effects increase (O’Hagan, 1979).

Low MAF and small sample sizes affect the AUC of all methods similarly (Fig. 2C—i, D—i). As MAF and sample size increase, the AUC improves and BTH and *dglm* have a greater AUC relative to the other methods (Fig. 2C—i, ii, iii, D—i, ii, iii). We note that CLS, across these ideal simulations, appears to have generally worse performance than Levene, Brown-Forsythe, BTH and *dglm*. In particular, the AUC for BTH and *dglm* are significantly greater than either Levene, Brown-Forsythe or CLS (Mann-Whitney U-test: *P *≤* *2.2 × 10^–^^16^ for all three, Fig. 2D—i), with an average precision 5% higher. Similarly, for higher mean effects (Fig. 2A—i, ii, iii) and large sample sizes (Fig. 2D—i, ii, iii), the relative performance of CLS deteriorates, with the AUC of CLS ≤ 0.036 smaller than the AUC of either Levene or Brown-Forsythe, and ≤ 0.12 smaller than the AUC of BTH and *dglm* (Fig. 2B—i).

### 3.2 Simulation results: ideal model, continuous covariates

When the covariate is continuous—such as age, BMI or the expected number of minor alleles for imputed genotypes—the Levene and Brown-Forsythe tests are no longer appropriate, as they assume categorical covariates. In this case, we compared our method with the CLS method, which allows a general covariate in the original linear regression and subsequent correlation test. We also applied Brown-Forsythe and Levene tests to the simulated data by rounding the continuous covariates to their nearest integer value. For imputed genotypes, this rounding process corresponds to setting the value of the variant to the most likely number of copies of the minor allele. This is an idealized imputation scenario (results on imputed genotypes below are much less straightforward), and we discourage this rounding approach with real data (Marchini and Howie, 2010).

**Table 1.** Different hypotheses tested in various data scenarios

Hypothesis	strong	weak
Null	*β* = 0 and log (*α*) = 0	*β* ≠ 0 and log (*α*) = 0
Alternative	*β* ≠ 0 and log (*α*) ≠ 0	*β* = 0 and log (*α*) ≠ 0

*Note*: The BTH model integrates over the mean effect size, *β*, testing the union of the weak and strong alternative hypotheses against the union of the weak and strong null hypotheses.

**Fig. 3.**
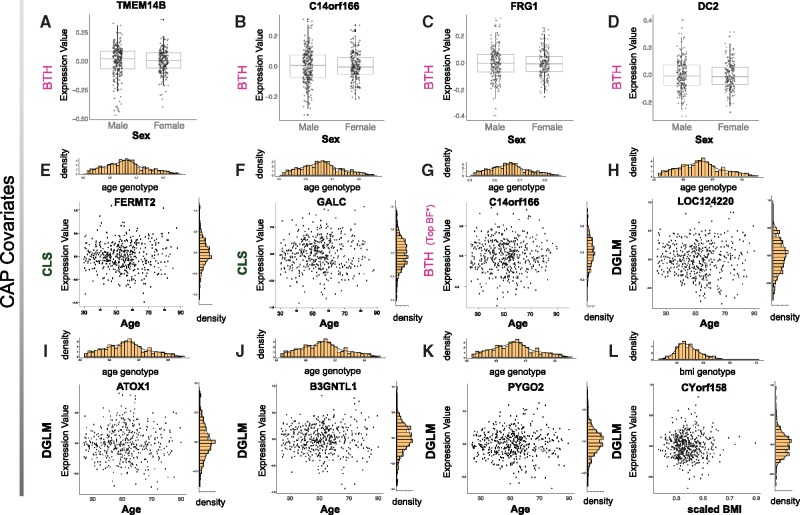
Variance controlling covariates uncovered by BTH and related tests in the CAP data (FDR ≤ 0.05). Panels **A**–**D**: genes with sex-dependent significant variance association according to BTH; Panels **E**, **F**: genes with significant age-dependent variance association according to CLS; Panel **G**: top gene with age-dependent variance association according to BTH; Panels **H**–**K**: genes with significant age-dependent variance association according to *dglm*; Panel **L**: gene with significant BMI-dependent variance association according to *dglm*

The results on the continuous covariate simulations echo the discrete simulation results. In particular, both BTH and *dglm* show uniform improvement in AUC across all of the simulations, considering increasing mean effects (Supplementary Fig. S1A), increasing variance effects (Supplementary Fig. S1B), increasing minor allele frequency (Supplementary Fig. S1C) and increasing sample size (Supplementary Fig. S1D). We note that, despite rounding the (idealized) imputed genotypes, Levene and Brown-Forsythe continue to perform better than CLS across the simulations.

### 3.3 Simulation results: non-ideal model, discrete covariates

Next, we explored quantitative traits simulated from four non-ideal heteroskedastic models with discrete covariates that are motivated by residual distributions often found in genomic analyses.

1. *Additive variance term:* a Gaussian distribution with an additive form of heteroskedasticity:$y_{i}∼N(\beta_{0}+\beta x_{i},\sigma^{2}+\alpha \cdot x_{i})$. We generated data with additive variance effects to ensure that our test is able to identify different functional forms of heteroskedasticity. Note that, in this scenario, the null hypothesis corresponds to *α* = 0; parameters in this category of simulation reflect this different null.
2. *Log Gaussian:* the log of the trait follows a Gaussian distribution: $y_{i}∼\;\text{exp}\;\left\{N\left(\beta_{0}+\beta x_{i},\sigma^{2}\alpha^{-x_{i}}\right)\right\}$. Microarray data are believed to have a log Gaussian distribution within gene, which motivates log transformations to those data (Irizarry *et al.*, 2003). Untransformed log normal data, however, will naturally appear heteroskedastic because of the correlation of mean and variance in the log Gaussian distribution.
3. *Gamma distributed data:* traits are generated from a gamma generalized linear model: $y_{i}∼Gamma\left(\mu_{i},1\right),\;\mu_{i}=\frac{1}{\beta_{0}+\beta x_{i}}$. While the exponential distribution is the continuous form of the Poisson distribution, the gamma distribution may be considered the continuous form of the negative binomial distribution, which is a discrete distribution with an additional variance parameter above the discrete Poisson distribution. Hence, we generate continuous data from the gamma distribution to simulate the continuous trait form of overdispersed Poisson counts, as might be found in mapped RNA-sequencing data (Marioni *et al.*, 2008; Pickrell *et al.*, 2010).
4. *Mixture of Gaussians:* traits are generated from a mixture of two Gaussian components, one heteroskedastic and one homoskedastic, with mixture parameter *λ* = 0.4: $y_{i}∼\lambda N(10,1)+(1-\lambda )N(\beta_{0}+\beta x_{i},\sigma^{2}\alpha^{-x_{i}})$. We expect bimodal Gaussian traits when, for example, there is an epistatic G×G or G×E interaction The presence of an epistatic term will correspond to a new mode in the distribution of the quantitative trait. For a binary interaction term this will correspond to a mixture of two distributions. For example, if there is a mean effect for female samples at a locus, but no corresponding mean effect for male samples, the quantitative trait will appear bimodal within genotype. We contrast this with the signature from a truly heteroskedastic effect, where we see an association with the variance of the trait distribution but do not see a mixture of distributions.

We quantified the relative performance of the tests using precision-recall curves as above; however, caution must be used here in interpreting the relative AUC. We consider four possibilities for the simulated mean and variance effects with respect to the statistical test we perform perform (Table 1):

- *Strong null:* the simulated mean effects *β* = 0 and the simulated variance effects log (*α*) = 0;
- *Weak null:* the simulated mean effects *β* ≠ 0 and the simulated variance effects log (*α*) = 0;
- *Weak alternative:* the simulated mean effects *β* = 0 and the simulated variance effects log (*α*) ≠ 0;
- *Strong alternative:* the simulated mean effects *β* ≠ 0 and the simulated variance effects log (*α*) ≠ 0.

These definitions become important when discussing the log Gaussian and gamma simulations: for both, the variance is a function of the mean, inducing an explicit relationship between the two. In other words, when there are mean effects, *β *≠ 0, this will present as variance effects in these tests. The BTH model integrates over the mean effect size, *β*, testing the union of the weak and strong alternative hypotheses against the union of the weak and strong null hypotheses. Moreover, in the permutations, we specifically remove variance effects while maintaining mean effects. These design decisions lead to different behavior of the test on these simulations from non-ideal scenarios.

For the non-ideal simulations, we simulated data both from the strong alternative (*β* ≠ 0, log (*α*) ≠ 0; Supplementary Fig. S2, first column) and the weak null (*β* ≠ 0, log (*α*) = 0; Supplementary Fig. S2, second column). The weak null simulation ideally will look like the null simulations; however, for the log Gaussian and gamma simulations, the tests differentiate the weak null and the strong null as an artifact of the data distribution. This phenomenon may be seen in the results by comparing the AUC of the strong alternative simulations with the weak alternative simulations: for the gamma simulations, the four tests have nearly identical AUCs regardless of the true value of the variance effects *α*. This suggests that the performance in the strong alternative simulations is due to mean effects. We verified this by considering simulations from the weak alternative [i.e. log (*α*) ≠ 0, *β* = 0], finding that all of the tests fail to detect signs of heteroskedasticity in the gamma simulations (Supplementary Fig. S2C). Similarly, in the untransformed log Gaussian simulations, test performance on the weak alternative scenario is close to that for the strong null (Supplementary Fig. S2B).

For the additive variance effects simulations and the bimodal distributed simulations, we find that the weak null simulations are appropriately unable to differentiate the weak null from the true null simulations (Supplementary Fig. S2—A i–iv; D i—iv). Moreover, for the bimodal distributed simulations, BTH and *dglm* had the most substantial gains in AUC relative to the other three methods, all of which had noticeably worse performance than in the ideal unimodal simulations. We further study departures from the ideal distributions below in the genomics applications.

To address the problem of distributional misspecification of the model, we developed a statistical classifier that takes as input the *x* and *y* vectors (covariates and traits, respectively) and returns the probability of each of seven distributions within and across groups for discrete covariates and across values for continuous covariates (Supplementary Figs S11, S12 and Tables S5–S6). Given a distribution classification for a particular covariate-trait test, we then suggest a specific data transformation to encourage a 0.5 recall for the weak null simulations (i.e. mean effects but no explicit variance effects). In particular, when the data appear to have a log Gaussian distribution, we suggest a log transformation (Supplementary Fig. S2B); when the data appear Gamma distributed, we suggest a mean-centered square root transformation (Supplementary Fig. S2C). We compared the transformed strong alternative simulations (*β*  ≠  0, log (*α*) ≠ 0; Supplementary Fig. S2, third column), and found that BTH and *dglm* uniformly had the largest AUC across the five methods. We also compared results on the transformed weak null simulations (*β*  ≠  0, log (*α*) = 0; Supplementary Fig. S2, fourth column). The transformation eliminates the mean effect discoveries in all but the gamma simulations (Supplementary Fig. S2C); in gamma simulations, variance effects are nearly removed across the five methods. We explore gamma-distributed data in the methylation analysis below.

### 3.4 1000 Genomes Project methylation study data

We applied BTH and the alternative tests for variance effects to a genome scale differential DNA methylation study (Heyn *et al.*, 2013) to find variance methylation QTLs (meQTLs). These data consist of DNA methylation levels at 485 577 CpG sites across the human genome using the Infinium HumanMethylation450 BeadChip platform (Illumina) in lymphoblastoid cell lines (LCLs) from 288 individuals—96 American with Northern and Western European ancestry, 96 Han Chinese and 96 Yoruban.

Following previous work, we removed CpG probes of poor quality or with common mutations. We used the *β* values from the methylation arrays at 406 021 CpG sites for analysis. Genotype information for these individuals are available from HumanHap550k and HumanHap650k genotype arrays (Illumina) at the GEO accession numbers GSE24260 (192 individuals) and GSE24274 (96 individuals). We removed eight individuals that did not have methylation data, and combined the genotypes from 280 individuals with 170 063 SNPs common to both genotype platforms and without missing data.

For each CpG site, we tested for association with cis-variants, defined as variants within 10KB of the CpG site. We evaluated the global FDR of our association results using a single permutation of the methylation data. Significance was assessed using a global FDR and FDR stratified by MAF. Using our distribution classifier, we found that most of the methylation level traits were gamma distributed (Supplementary Tables S2 and S3).

BTH does not find any significant variant-mediated associations between genetic variants and methylation levels at CpG sites at a global FDR of 0.05 and a MAF-stratified FDR of 0.05 (Supplementary Table S1).

In contrast, *dglm* identified three significant associations, CLS identified 549 significant associations, and the Levene test identified 878 significant associations (global FDR ≤ 0.05, Supplementary Table S1). However, in these discoveries, a large majority of the distribution of methylation levels were found to be either bimodal or multimodal, with unimodal traits making up 8.95% of the discoveries from CLS and 0.38% of the discoveries from the Levene test (Supplementary Figs S3–S6). We hypothesize that the general bimodal distribution of methylation values with respect to genotype is due to ubiquitous epistatic effects. BTH and *dglm*, on the other hand, are robust to bimodal deviations from the unimodal Gaussian distribution, and do not detect these candidates for epistatic effects at an FDR ≤ 0.05.

We tested for variance meQTLs without transforming the methylation data under the assumption that a single variant will not have both mean and variance effects on methylation levels at a single CpG site. BTH detected no variance associations, meaning that false positives due to confounding effects were not apparent in the data. Had there been discoveries for BTH, we would have repeated the test with the appropriately transformed data using a square root transform.

### 3.5 Cardiovascular and Pharmacogenetics (CAP) study

We applied BTH to test for variance effects between imputed genotypes and gene expression levels from the Cardiovascular and Pharmacogenetics (CAP) study.

Gene expression values for 10 195 genes in lymphoblastoid cell lines (LCLs) from 480 Caucasian individuals were assayed on human microarray platforms. Genotypes were assayed using genotyping arrays and subsequently imputed using IMPUTE2 to yield 33 386 856 total markers across the 22 autosomal chromosomes. We removed variants with MAF below 0.05.

The preprocessing of gene expression data for testing of variance eQTLs is somewhat different than the preprocessing for mean effect eQTLs. To test for variance eQTLs, we log transform the microarray gene expression data so they do not have a log normal distribution, we control for outliers, and we control for known (directly measured) and unknown (inferred) confounders (Methods). After preprocessing the genotype and gene expression data, we performed association mapping between each gene and the cis-SNPs local to that gene; here, cis-SNPs are defined to be ≤ 200 Kb from the gene transcription start or end site. There were 9862 genes with at least one cis-SNP in these data, and, on average, each gene had 847 cis-SNPs. We computed the test statistic for the putative association between each cis-SNP gene pair with these processed gene expression data.

As in the methylation study data, BTH finds no significant associations in our permutation-based testing method. While the *dglm* and CLS tests report *P*-values for each association (it is therefore possible to assign a *P*-value cutoff to identify significant results for each method), we chose to base the significance of the *dglm* and CLS tests on our previously described FDR method using permutations. We calibrate the FDR for each test according to this same permutation strategy for consistency. When subsetting the statistical tests by MAF range, BTH uncovers six associations, including a locus regulating variance of the gene *DIS3L*, recently identified as a possible risk factor for myocardial infarction (Lee *et al.*, 2017), and a locus regulating the variance of the gene *MAP2K1*, involved in cardiac signaling (Krysiak *et al.*, 2018; Sheikh *et al.*, 2008) (Supplementary Table S4, Fig. S8).

### 3.6 Variance effects of CAP study covariates

Next, we applied BTH to test for a heteroskedastic relationship between gene expression levels and known covariates collected on the individuals enrolled in the CAP study. In particular, we considered sample age, sex, BMI and smoking status. For non-binary covariates (age and BMI), we normalized the values—dividing each covariate by its maximum such that each covariate had a maximum value of one—for stability of parameter estimation; this does not change the interpretation of our results. Both sex and smoking status are binary covariates, so the application of BTH is equivalent to testing for differential variance across the binary covariate. Overall, these data contain 46% females and 87% non-smokers.

In these data, we found four associations using BTH, six associations using *dglm* and 17 associations using CLS (FDR ≤ 0.05; 3, Supplementary Table S3). All four BTH associations corresponded to sex specific variance control. Four of the five *dglm* associations corresponded to age specific variance control, and one to BMI specific variance control. CLS identified 15 significant age associations and two significant sex associations (Fig. 3). In particular, BTH discovers variance effects of sex in transmembrane protein *TMEM14B* and in the protein coding genes *DC2* (or *OSTC*), *C14orf166* and *FRG1*. Among these, of interest is the gene *C14orf166* which is involved in viral replication in the case of infection with influenza virus A (Rodriguez *et al.*, 2011). Similarly, CLS discovers variance effects of age in the genes *FERMT2* and *GALC*. The gene *FERMT2* or *Kindlin-2* is particularly known to interact with beta catenin and is associated with the integrin signaling pathway, cell adhesion and mutagenesis (Meller *et al.*, 2015). Lastly, the genes discovered by *dglm* are protein coding genes, among which *ATOX1* is a known copper metallochaperone protein, and *B3GNTL1* is involved in transferase activity and in the transfer of glycosyl groups. While further research is needed to validate the role of these genes, their discovery is unsurprising given the existing evidence across various species for genetic control of phenotypic variation in the context of the obesity, metabolic or immune functions of evolutionary conserved targets (Ansel *et al.*, 2008; Hill and Mulder, 2010; Queitsch *et al.*, 2002; Yang *et al.*, 2012).

## 4 Materials and methods

### 4.1 Bayesian test for heteroskedasticity (BTH)

The observed data are two vectors, $y\in R^{n}$ (quantitative trait) and $x\in R^{n}$ (covariate). For each sample i={1,…n}, we model the quantitative trait as $y_{i}∼\beta_{0}+\beta x_{i}+N(0,\sigma^{2}\alpha^{-x_{i}})$, with appropriate priors (Results). We set the hyperparameters of the heteroskedastic parameter as follows: *x*_0_ = 0, centering the Cauchy distribution at 0, *ν*  =  5, $\theta_{1}=1.,\;\theta_{2}=2$ and *γ*  =  1.

BTH computes the likelihood of the alternative hypothesis versus the likelihood of the null hypothesis (Kass and Raftery, 1995):

- *H*_0_, the null hypothesis where *α* = 1 or, equivalently, log (*α*) = 0;
- *H_A_*, the alternative hypothesis where *α* ≠ 1 or, equivalently, log (*α*) ≠ 0.

BFs are computed by integrating over uncertainty in each of the model parameters *β*_0_, *σ*^2^ and *α* after computing a closed-form integral over the effect size *β* using multivariate Laplace approximations (Rue *et al.*, 2009) (Supplementary Methods). As is common in Bayesian analysis of genomic studies, we report the log_10_ transformed BFs (Stephens and Balding, 2009).

### 4.2 FDR calibration

Global false discovery rate (FDR) of the log BFs was quantified using permutations. To do this, we developed a permutation that preserved the mean effects but removed any variance effects. In particular, for trait $y\in R^{n}$, we computed a mean-effect-preserving transformation as follows. We fit a linear regression model using generalized least squares and computed residuals $r_{i}=y_{i}-\beta_{gls}x_{i}$ for each sample *i*. We then randomly permuted the sample indices on *r_i_*, $r_{\pi (i)}$, checking that the mean effects of $r_{\pi (i)}$ versus *x_i_* are not statistically different than zero. Finally, we set the permuted value ${\tilde{y}}_{i}=\beta_{gls}x_{i}+r_{\pi (i)}$.

Global FDR calibration was performed after computing the unpermuted and permuted BFs, $BF^{\left(0\right)}=BF(x,y)$ and $BF^{\left(\pi \right)}=BF(x,\tilde{y})$. For a BF threshold *d*, true positives (TP) and false positives (FP) are estimated using these BFs as $\hat{TP}=\#\{j:|BF_{j}^{\left(0\right)}|>d\}$ and $\hat{FP}(d)+\hat{FP}(d)=\#\{j:|BF_{j}^{\left(\pi \right)}|>d\}$ respectively. Thus, the estimated FDR at threshold *d* is computed as $\hat{FDR}(d)=\frac{\hat{FP}(d)}{\hat{TP}(d)+\hat{FP}(d)}$. For a specific FDR threshold, the calibrated threshold *d_FDR_* is computed from the data, and the pairs (*x*, *y*) with $BF(x,y)>d_{FDR}$ are reported.

### 4.3 Levene, Brown-Forsythe, CLS and *dglm*

The Levene, Brown-Forsythe, *dglm* and CLS tests were implemented and applied for comparison with BTH. The Brown-Forsythe and Levene tests both belong to the general Levene family of tests for equality of variance across *k* subgroups. For *n* samples corresponding to categorical covariate $x\in {\left\{1,2,\ldots k\right\}}^{n}$, the trait $y\in R^{n}$ is modeled as $y_{i}∼N(\beta_{0}+\beta x_{i},\sigma_{x_{i}}^{2})$. The null hypothesis *H*_0_ corresponds to equal variances across subgroups $\sigma_{j}^{2}=\sigma_{\ell }^{2}$ for all j,ℓ∈{1,2,…,k}. The alternative hypothesis *H_A_* corresponds to unequal variances across subgroups $\sigma_{j}^{2}\neq \sigma_{\ell }^{2}$ for at least one pair j≠ℓ, j,ℓ∈{1,2,…,k}. For each subgroup t∈{1,2,…,k}, let *n_t_* be the number of samples in **x**. The corresponding vectors $\omega_{t}\in R^{n_{t}},\;\omega =\{y_{i}|x_{i}=t\}$, are a partition of the trait values **y**. For each *t*, let *ω_t_*(*s*) be entry *s* of the ω vector. The mean of the trait values within group *t* is the mean of the entries $\omega_{t}(s),\;s\in \{1,2,\ldots ,n_{t}\}$, which we denote by ${\bar{\omega }}_{t}$. Then,

(3) $$W=\frac{(n-k)\sum_{t=1}^{k}{\left({\bar{z}}_{t}-\bar{z}\right)}^{2}}{(k-1)\sum_{t=1}^{k}\sum_{s=1}^{n_{t}}{\left(z_{ts}-{\bar{z}}_{t}\right)}^{2}},$$

where $z_{ts}=|\omega_{t}(s)-{\bar{\omega }}_{t}|,\;{\bar{z}}_{t}=\frac{1}{n_{t}}\sum_{s=1}^{n_{t}}z_{ts}$ is the mean over the values *z_ts_*, and $\bar{z}=\frac{1}{n}\sum_{t=1}^{t=k}\sum_{s=1}^{n_{t}}z_{ts}$ is the overall mean over the entries of the vector trait *y*. When each ${\bar{\omega }}_{t}$ is the median of *ω_t_*(*s*) values instead of their mean, this becomes the Brown-Forsythe test. Significance and global FDR were computed based on permutations as with BTH, replacing BFs with *P*-values.

The CLS test was implemented by computing residuals $r_{i}=y_{i}-{\hat{\beta }}_{0}-\hat{\beta }x_{i}$, where ${\hat{\beta }}_{0}$ and $\hat{\beta }$ were fit using generalized least squares, modeling the trait *y_i_* conditional on the genotype *x_i_* for each individual *i* using linear regression. The Spearman rank correlation test between the squared residuals, $r_{i}^{2}={\left(y_{i}-({\hat{\beta }}_{0}+{\hat{\beta }}_{i}x_{i})\right)}^{2}$ and the genotypes, *x_i_*, correspond to a test for variance QTLs. This implementation of CLS followed the description in prior work (Brown *et al.*, 2014).

Finally, *dglm* is a statistical test in which both the mean and the variance are estimated as generalized linear models (Dunn and Smyth, 2012; Verbyla and Smyth, 1998), where the dispersion or variance parameter is generally modeled through a gamma regression. The estimation of the mean and variance parameters is performed iteratively, and *P*-values are computed using a *χ*-square test using R package *dglm* (Dunn and Smyth, 2012). Significance and global FDR were computed using *P*-values based on permutations as with the other methods. In particular, we applied *dglm* with appropriate overdispersion options and links from Gaussian and gamma families when appropriate (gamma simulation). While *dglm* performs similarly to BTH in simulation, its reliance on *glm* results in convergence problems of the iteratively reweighted least squares procedure, thus making it unreliable when scaling to large applications.

### 4.4 Regression distribution classifier

We trained a random forest classifier [RandomForest in scikit-learn (Pedregosa *et al.*, 2011), version 0.16.1] to distinguish between six possible departures from the ideal heteroskedastic model. For each distribution class (the BTH model, additive variance model, exponential mean model, exponential residual model, log Gaussian, gamma and bimodal models; see Supplemental Methods for descriptions) and for four parameter configurations (scenarios *α*  ≠  0 and *β*  ≠  0, *α*  ≠  0 and *β*  =  0, *α*  =  0 and *β*  ≠  0, and *α*  =  0 and *β*  =  0), we generated 50 samples of observed data from those six models. We computed one-sided Kolmogorov-Smirnov (KS) statistics between each of these samples and 100 samples, with matched sample sizes, generated from 79 distinct probability density functions with matched mean and variance. Thus, every sample is represented as a point in a 79-dimensional feature space. Performance of the RF classifier was evaluated using five-fold cross validation. The performance of the classifier was quantified using a precision recall curve on cross-validated simulation data (Supplementary Fig. S11–S12).

### 4.5 HapMap phase 2 methylation study data

Genotype information for the HapMap phase 2 methylation study data are available from HumanHap550k and HumanHap650k genotype arrays (Illumina) at the GEO accession numbers GSE24260 (Kalari *et al.*, 2010) (192 individuals) and GSE24274 (Niu *et al.*, 2010) (96 individuals). We removed eight individuals that did not have methylation data, and combined the genotypes from 280 individuals with 170 063 variants common to both genotype platforms and without missing data. For each CpG site, we tested for association with cis-variants, defined as variants within 10Kb of the CpG site (Bell *et al.*, 2011; Heyn *et al.*, 2013). We evaluated the global FDR of our association results using a single permutation of the methylation data. Significance was assessed using a global FDR and FDR stratified by MAF (Sun *et al.*, 2006) (Supplementary Table S1). Using our distribution classifier, we found that most of the methylation level traits were gamma distributed (Supplementary Tables S2 and S3).

Processed DNA methylation data using the Infinium *HumanMethylation450* BeadChip platform were downloaded from the Gene Expression Omnibus (GEO), accession number GSE36369 (Heyn *et al.*, 2013) on August 6, 2015. We used methylation data for 280 individuals for whom genotypes were available. Genotypes spanning 166 947 common genetic variants were obtained from DNA array Human Variation Panel studies (Kalari *et al.*, 2010; Niu *et al.*, 2010), accession numbers GSE24260 and GSE24274, which assayed genotypes using Illumina 550K and Illumina 650K arrays, respectively. We filtered poor quality CpG probes by removing methylation sites where 90% of the samples at that site are hypo- or hyper-methylated (<0.02 or >0.98 methylated, respectively). From a total of 54 750 total CpG probes, we filter 2112 probes to yield 52 638 probes for association tests with genotypes.

### 4.6 Cardiovascular and Pharmacogenetics (CAP) study

Gene expression levels from 10 195 genes in lymphoblastoid cell lines (LCLs) created from 480 genotyped individuals were downloaded from the Gene Expression Omnibus (GSE36868). Genotypes for 387 514 variants and eight other covariates were available through dbGaP (Study Accession phs000481.v1.p1) (Mangravite *et al.*, 2013). We processed the raw gene expression data as follows.

1. *Log transform:* A log _2_ transformation was applied to each entry of the gene expression matrix;
2. *Control for latent population structure:* We computed the first two principal components *x_PC_*_1_, *x_PC_*_2_ of the genotype matrix via singular value decomposition (SVD).
3. *Control for known covariates; mean center:* For each vector *y_j_* in matrix **Y**, corresponding to single gene *j* across all *n* samples, a linear model $y_{j}=\lambda_{0}+\lambda_{age}\cdot x_{age}+\lambda_{sex}\cdot x_{sex}+\lambda_{batch}\cdot x_{batch}+\lambda_{PC1}\cdot x_{PC1}+\lambda_{PC2}\cdot x_{PC2}$ was fitted to account for variation in gene expression due to sample age, sex, batch, two PCs from the gene expression matrix, and two genotype PCs, using generalized least squares. Mean-centered residuals $r_{j}=y_{j}-{\hat{\lambda }}_{0}-{\hat{\lambda }}_{age}\cdot x_{age}-{\hat{\lambda }}_{sex}\cdot x_{sex}-{\hat{\lambda }}_{batch}\cdot x_{batch}-{\hat{\lambda }}_{PC1}\cdot x_{PC1}-{\hat{\lambda }}_{PC2}\cdot x_{PC2}$ were computed. Concatenating the *r_j_* vectors gives us the normalized expression matrix.
4. *Control for unknown covariates:* We computed the first two PCs of the normalized expression matrix using SVD. We used linear regression as in the previous step to control for the linear effects of these two PCs in the normalized gene expression matrix.

The resulting matrix is the processed gene expression matrix. After empirical quantile normalization (Brown *et al.*, 2014), each gene has exactly the same distribution across all samples, and a visual analysis of a QQ-Plot confirms the empirical distribution deviates little from a normal distribution (Supplementary Fig. S7).

After preprocessing the genotype and gene expression data, we performed association mapping between each gene and the cis-variants local to that gene; here, cis-variants are defined to be ≤ 200 Kb from the gene transcription start or end site (Pickrell *et al.*, 2010). There were 9862 genes with at least one cis-variant in these data, and, on average, each gene had 847 cis-variants. We computed the test statistic for the putative association between each cis-variant gene pair with these processed gene expression data (Pickrell *et al.*, 2010).

## 5 Discussion

We presented a Bayesian test for heteroskedasticity (BTH) that allows for continuous covariates and incorporates uncertainty in estimates of mean and variance effects of covariates to robustly test for variance QTLs and QTCs. We evaluated our approach and compared it to state-of-the-art methods on extensive simulated datasets conforming to, and in violation of, the assumptions in our model. We described a prescriptive procedure to ensure a well-powered application of our model to diverse genomic and epigenetic study data. Although we are mainly focused on variance effects of genotype on quantitative traits, this approach may be used broadly in testing for heteroskedastic associations, and we show this application by discovering meaningful associations between non-genetic covariates and gene expression data.

In the Results, we note that BTH and *dglm* are more conservative and less sensitive to multi-modal distributions than both CLS and the Levene test, as we showed in the multi-modal simulation studies and through spurious results from CLS and the Levene test in the methylation data. In scenarios where the data are close in distributional form to the modeling assumptions, as in the gene expression data, BTH finds similar numbers of associations as CLS. While our findings show that *dglm* outperforms the Levene, Brown-Forsythe and CLS tests in multiple simulation settings, its iterative approach to fitting often fails to converge because of sensitivity to step size. This shortcoming makes *dglm* cumbersome for genome-wide variance eQTL analysis, and we recommend BTH as a more reliable alternative. While BTH is three times slower than *dglm*, which often converges in under 1s on a machine with Intel Core running at 2.2 GHz, BTH is easily parallelized for genome-wide associations.

The lack of results from BTH in the methylation data raise an important discussion point. In particular, the signature of gene × gene or gene × environment epistatic interactions may show up as a bimodal distribution of the trait: consider the distribution of a trait that has an eQTL with mean effect in women but not in men. We note that our statistical test was robust to deviations from unimodality, but CLS and Levene were not, making the purpose of these tests somewhat orthogonal. Thus, to identify candidate epistatic associations, CLS and Levene are the appropriate methods to use; on the other hand, to identify variance effects, our method is superior in terms of statistical power. We also hypothesize that the permutations that are used for these tests, while appropriate, lead to conservative estimates of FDR, which impacted all of the statistical tests calibrated using permutations.

The lack of power in the variance QTL studies was clear: unlike mean effects, we found no variance effects of genetic variants on methylation, and six significant variance effects on gene expression levels. We note that, if *n* samples are well powered to detect mean effects of a certain size, to detect comparable variance effects at the same precision, a sample of *O*(*n*^2^) is needed; thus additional samples will facilitate finding these effects. We also propose that these measurements of cellular traits are inappropriate candidates for variance QTLs because the variance effects will not be across individuals but instead across cells within an individual, as shown in previous studies (Wills *et al.*, 2013). In particular, a variance QTL impacts the variability of gene expression or methylation levels across the sample cells. The bulk measurement of these cellular traits, however, are performed on tens of thousands of cells, and quantify the average expression levels across those cells. Thus, in order to identify variance QTLs, different types of data must be considered such as single cell RNA-sequencing data (Wills *et al.*, 2013) or resampled RNA-sequencing data to estimate within-sample variance (Auer and Doerge, 2010).

Our BTH framework improves on existing methods with a flexible modeling framework, integrating over uncertainty, and fast robust statistical inference, leading to improved power to detect heteroskedastic associations. Identifying heteroskedastic associations in quantitative traits will augment our catalog of quantitative trait regulation and lead to an improved understanding of the mechanisms of genetic control over phenotypes.

## Supplementary Material

Supplementary Figures

Supplementary Tables

Supplementary Text

## References

[bty565-B1] Ansel J. et al (2008) Cell-to-cell stochastic variation in gene expression is a complex genetic trait. PLoS Genet., 4, e1000049. 18404214 10.1371/journal.pgen.1000049 PMC2289839

[bty565-B3] Auer P.L., Doerge R. (2010) Statistical design and analysis of RNA sequencing data. Genetics, 185, 405–416. 20439781 10.1534/genetics.110.114983 PMC2881125

[bty565-B4] Ayroles J.F. et al (2015) Behavioral idiosyncrasy reveals genetic control of phenotypic variability. Proc. Natl. Acad. Sci. USA, 112, 6706–6711. 25953335 10.1073/pnas.1503830112 PMC4450409

[bty565-B5] Bartlett M.S. (1937) Properties of sufficiency and statistical tests. Proc. R. Soc. Lond. Ser. A Math. Phys. Sci., 160, 268–282.

[bty565-B6] Battle A. et al (2014) Characterizing the genetic basis of transcriptome diversity through RNA-sequencing of 922 individuals. Genome Res., 24, 14–24. 24092820 10.1101/gr.155192.113 PMC3875855

[bty565-B7] Bell J.T. et al (2011) DNA methylation patterns associate with genetic and gene expression variation in HapMap cell lines. Genome Biol., 12, R10. 21251332 10.1186/gb-2011-12-1-r10 PMC3091299

[bty565-B8] Brown A.A. et al (2014) Genetic interactions affecting human gene expression identified by variance association mapping. eLife, 3, e01381. 24771767 10.7554/eLife.01381 PMC4017648

[bty565-B9] Brown M.B., Forsythe A.B. (1974) The small sample behavior of some statistics which test the equality of several means. Technometrics, 16, 129–132.

[bty565-B10] Cao Y. et al (2014) A versatile omnibus test for detecting mean and variance heterogeneity. Genet. Epidemiol., 38, 51–59. 24482837 10.1002/gepi.21778 PMC4019404

[bty565-B11] Cao Y. et al (2015) A family-based joint test for mean and variance heterogeneity for quantitative traits. Ann. Hum. Genet., 79, 46–56. 25393880 10.1111/ahg.12089 PMC4275359

[bty565-B12] Dunn P.K., Smyth G.K. (2012) dglm: Double Generalized Linear Models. R Package Version, 1.

[bty565-B13] Gibson G., Wagner G. (2000) Canalization in evolutionary genetics: a stabilizing theory? Bioessays, 22, 372–380. 10723034 10.1002/(SICI)1521-1878(200004)22:4<372::AID-BIES7>3.0.CO;2-J

[bty565-B2] GTEx Consortium (2017) Genetic effects on gene expression across human tissues. Nature, 550, 204–213. 29022597 10.1038/nature24277 PMC5776756

[bty565-B14] Heyn H. et al (2013) DNA methylation contributes to natural human variation. Genome Res., 23, 1363–1372. 23908385 10.1101/gr.154187.112 PMC3759714

[bty565-B15] Hill W.G., Mulder H.A. (2010) Genetic analysis of environmental variation. Genet. Res., 92, 381–395. 10.1017/S0016672310000546 21429270

[bty565-B16] Howie B.N. et al (2009) A flexible and accurate genotype imputation method for the next generation of genome-wide association studies. PLoS Genet., 5, e1000529. 19543373 10.1371/journal.pgen.1000529 PMC2689936

[bty565-B17] Irizarry R.A. et al (2003) Exploration, normalization, and summaries of high density oligonucleotide array probe level data. Biostatistics, 4, 249–264. 12925520 10.1093/biostatistics/4.2.249

[bty565-B18] Kalari K.R. et al (2010) Copy number variation and cytidine analogue cytotoxicity: a genome-wide association approach. BMC Genomics, 11, 357. 20525348 10.1186/1471-2164-11-357 PMC2894803

[bty565-B19] Kass R.E., Raftery A.E. (1995) Bayes factors. J. Am. Stat. Assoc., 90, 773–795.

[bty565-B20] Krysiak J. et al (2018) Protein phosphatase 5 regulates titin phosphorylation and function at a sarcomere-associated mechanosensor complex in cardiomyocytes. Nat. Commun., 9, 262. 29343782 10.1038/s41467-017-02483-3 PMC5772059

[bty565-B21] Lee J.-Y. et al (2017) Genome-based exome sequencing analysis identifies GYG1, DIS3L and DDRGK1 are associated with myocardial infarction in Koreans. J. Genet., 96, 1041–1046. 29321365 10.1007/s12041-017-0854-z

[bty565-B22] Levene H. (1961) Robust tests for equality of variances. *Contributions to Probability and Statistics. Essays in Honor of Harold Hotelling*, pp. 279–292.

[bty565-B23] Mangravite L.M. et al (2013) A statin-dependent QTL for *GATM* expression is associated with statin-induced myopathy. Nature, 502, 377–380. 23995691 10.1038/nature12508 PMC3933266

[bty565-B24] Marchini J., Howie B. (2010) Genotype imputation for genome-wide association studies. Nat. Rev. Genet., 11, 499–511. 20517342 10.1038/nrg2796

[bty565-B25] Marioni J.C. et al (2008) RNA-seq: an assessment of technical reproducibility and comparison with gene expression arrays. Genome Res., 18, 1509–1517. 18550803 10.1101/gr.079558.108 PMC2527709

[bty565-B26] Meller J. et al (2015) Emergence and subsequent functional specialization of kindlins during evolution of cell adhesiveness. Mol. Biol. Cell, 26, 786–796. 25540429 10.1091/mbc.E14-08-1294 PMC4325847

[bty565-B27] Metzger B.P. et al (2015) Selection on noise constrains variation in a eukaryotic promoter. Nature, 521, 344. 25778704 10.1038/nature14244 PMC4455047

[bty565-B28] Nachman M.W. et al (2003) The genetic basis of adaptive melanism in pocket mice. Proc. Natl. Acad. Sci. USA, 100, 5268–5273. 12704245 10.1073/pnas.0431157100 PMC154334

[bty565-B29] Nelson M.R. et al (2012) An abundance of rare functional variants in 202 drug target genes sequenced in 14, 002 people. Science, 337, 100–104. 22604722 10.1126/science.1217876 PMC4319976

[bty565-B30] Niu N. et al (2010) Radiation pharmacogenomics: a genome-wide association approach to identify radiation response biomarkers using human lymphoblastoid cell lines. Genome Res., 20, 1482–1492. 20923822 10.1101/gr.107672.110 PMC2963812

[bty565-B31] O’Hagan A. (1979) On outlier rejection phenomena in Bayes inference. J. R. Stat. Soc. Ser. B (Methodological), 358–367.

[bty565-B32] Paré G. et al (2010) On the use of variance per genotype as a tool to identify quantitative trait interaction effects: a report from the Women’s Genome Health Study. PLoS Genet., 6, e1000981. 20585554 10.1371/journal.pgen.1000981 PMC2887471

[bty565-B33] Pedregosa F. et al (2011) Scikit-learn: machine learning in Python. J. Mach. Learn. Res., 12, 2825–2830.

[bty565-B34] Pickrell J.K. et al (2010) Understanding mechanisms underlying human gene expression variation with RNA sequencing. Nature, 464, 768–772. 20220758 10.1038/nature08872 PMC3089435

[bty565-B35] Queitsch C. et al (2002) Hsp90 as a capacitor of phenotypic variation. Nature, 417, 618–624. 12050657 10.1038/nature749

[bty565-B36] Rodriguez A. et al (2011) Cellular human cle/c14orf166 protein interacts with influenza virus polymerase and is required for viral replication. J. Virol., 85, 12062–12066. 21900157 10.1128/JVI.00684-11 PMC3209284

[bty565-B37] Rönnegård L., Valdar W. (2011) Detecting major genetic loci controlling phenotypic variability in experimental crosses. Genetics, 188, 435–447. 21467569 10.1534/genetics.111.127068 PMC3122324

[bty565-B38] Rue H. et al (2009) Approximate Bayesian inference for latent Gaussian models by using integrated nested Laplace approximations. J. R. Stat. Soc. Ser. B (Stat. Methodol.), 71, 319–392.

[bty565-B39] Ruiz-Cárdenas R. et al (2012) Direct fitting of dynamic models using integrated nested Laplace approximations – INLA. Comput. Stat. Data Anal., 56, 1808–1828.

[bty565-B40] Salomé P.A. et al (2011) Genetic architecture of flowering-time variation in *Arabidopsis thaliana*. Genetics, 188, 421–433. 21406681 10.1534/genetics.111.126607 PMC3122318

[bty565-B41] Savolainen O. et al (2013) Ecological genomics of local adaptation. Nat. Rev. Genet., 14, 807–820. 24136507 10.1038/nrg3522

[bty565-B42] Schultz B.B. (1985) Levene’s test for relative variation. Syst. Biol., 34, 449–456.

[bty565-B43] Sheikh F. et al (2008) An fhl1-containing complex within the cardiomyocyte sarcomere mediates hypertrophic biomechanical stress responses in mice. J. Clin. Investig., 118, 3870–3880. 19033658 10.1172/JCI34472 PMC2575833

[bty565-B44] Shen X. et al (2012) Inheritance beyond plain heritability: variance-controlling genes in *Arabidopsis thaliana*. PLoS Genet., 8, e1002839. 22876191 10.1371/journal.pgen.1002839 PMC3410891

[bty565-B46] Soave D., Sun L. (2017) A generalized Levene’s scale test for variance heterogeneity in the presence of sample correlation and group uncertainty. Biometrics, 73, 960–971. 28099998 10.1111/biom.12651

[bty565-B47] Stephens M., Balding D.J. (2009) Bayesian statistical methods for genetic association studies. Nat. Rev. Genet., 10, 681–690. 19763151 10.1038/nrg2615

[bty565-B48] Stranger B.E. et al (2007) Relative impact of nucleotide and copy number variation on gene expression phenotypes. Science, 315, 848–853. 17289997 10.1126/science.1136678 PMC2665772

[bty565-B49] Struchalin M. et al (2012) An R package ‘VariABEL’ for genome-wide searching of potentially interacting loci by testing genotypic variance heterogeneity. BMC Genetics, 13, 4. 22272569 10.1186/1471-2156-13-4 PMC3398297

[bty565-B50] Sun L. et al (2006) Stratified false discovery control for large-scale hypothesis testing with application to genome-wide association studies. Genet. Epidemiol., 30, 519–530. 16800000 10.1002/gepi.20164

[bty565-B51] Verbyla A., Smyth G. (1998) Double generalized linear models: approximate residual maximum likelihood and diagnostics. Technical report, Research report, Department of Statistics, University of Adelaide.

[bty565-B52] Wang K. et al (2017) Classification of common human diseases derived from shared genetic and environmental determinants. Nat. Genet., 49, 1319. 28783162 10.1038/ng.3931 PMC5577363

[bty565-B53] Wills Q.F. et al (2013) Single-cell gene expression analysis reveals genetic associations masked in whole-tissue experiments. Nat. Biotechnol., 31, 748–752. 23873083 10.1038/nbt.2642

[bty565-B54] Yang J. et al (2012) FTO genotype is associated with phenotypic variability of body mass index. Nature, 490, 267–272. 22982992 10.1038/nature11401 PMC3564953

[bty565-B55] Zeggini E. et al (2007) Replication of genome-wide association signals in UK samples reveals risk loci for type 2 diabetes. Science, 316, 1336–1341. 17463249 10.1126/science.1142364 PMC3772310

