# Supplementary material for: Statistical tests for detecting variance effects in quantitative trait studies
Source: Bioinformatics. 2018 Jul 6;35(2):200–10. doi: 10.1093/bioinformatics/bty565 (PMC6330007; doi:10.1093/bioinformatics/bty565)
Supplement: Supplementary Figures [file bty565_supplementary_figures.pdf]

Supplementary figures for: Evaluation of statistical tests for detecting variance effects and a Bayesian approach to heteroskedasticity

Bianca Dumitrascu[1], Gregory Darnell[1], Julien Ayroles[1,2] & Barbara E Engelhardt[3,4]

[1]*Lewis-Sigler Institute, Princeton University.*

[2]*Department of Ecology and Evolutionary Biology, Princeton University.*

[3]*Department of Computer Science, Princeton University.*

[4]*Center for Statistics and Machine Learning, Princeton University.*

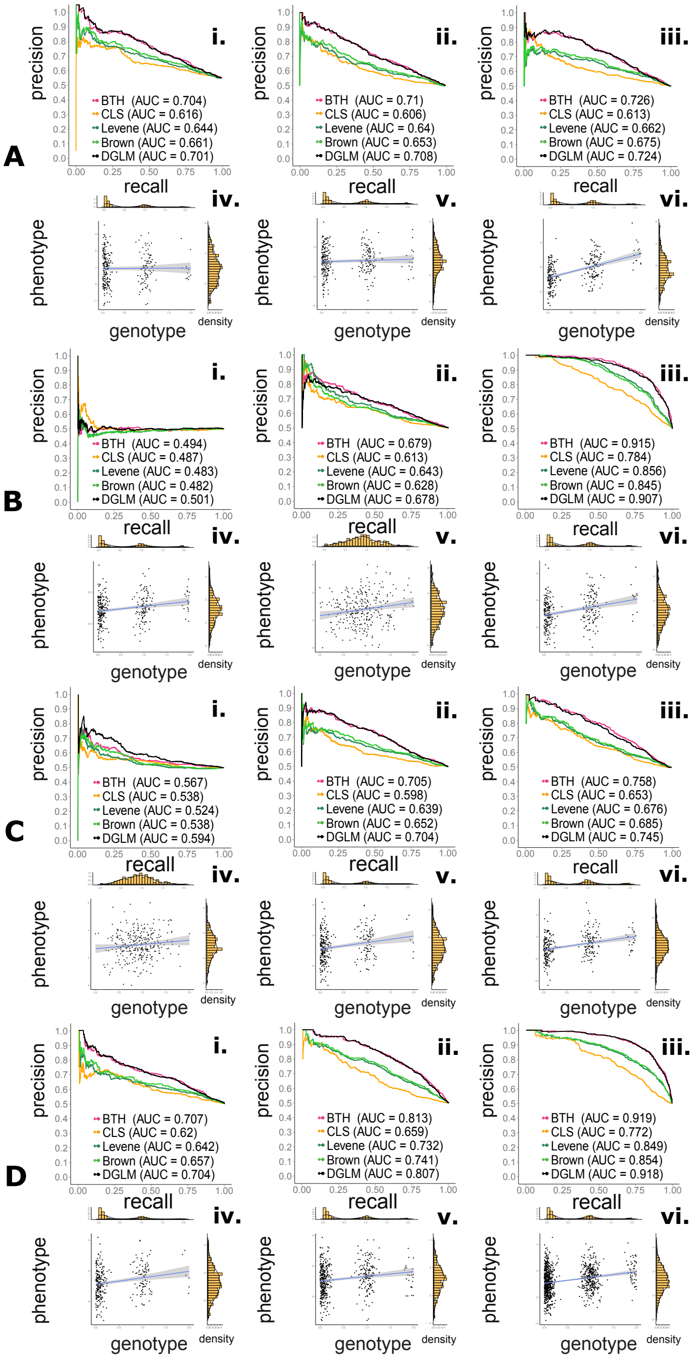

Figure 1: Precision-recall curves comparing performance of BTH versus CLS, Levene, and Brown-Forsythe and corresponding scatter plots of one example of this scenario. Panel A: increasing the mean effects: $\pi_{maf} = 0.2$, $n = 300$, $\beta \in \{0, 0.2, 1\}$, $\log(\alpha) = 0.1$; Panel B: increasing the variance effects: $\pi_{maf} = 0.2$, $n = 300$, $\beta = 0.5$, $\log(\alpha) \in \{0, -0.1, -0.2\}$; Panel C: increasing the minor allele frequency: $\pi_{maf} \in \{0.05, 0.2, 0.3\}$, $n = 300$, $\beta = 0.5$, $\log(\alpha) = 0.1$; Panel D: increasing the sample size: $\pi_{maf} = 0.2$, $n \in 300, 500, 1000$, $\beta = 0.5$, $\log(\alpha) = 0.1$.

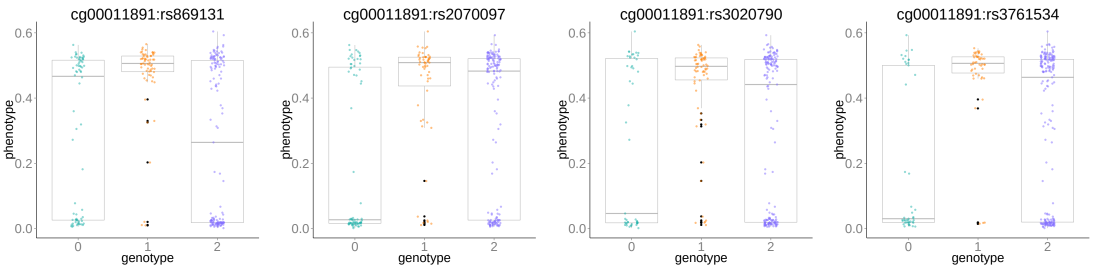

Figure 2: Examples of bi-modality among significant hits by Levene.

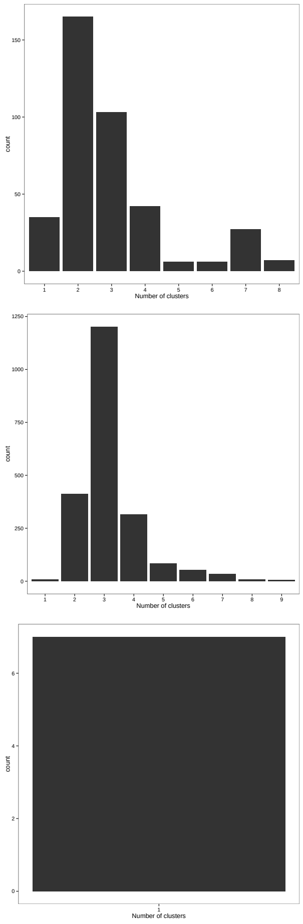

Figure 3: Results from methylation data.

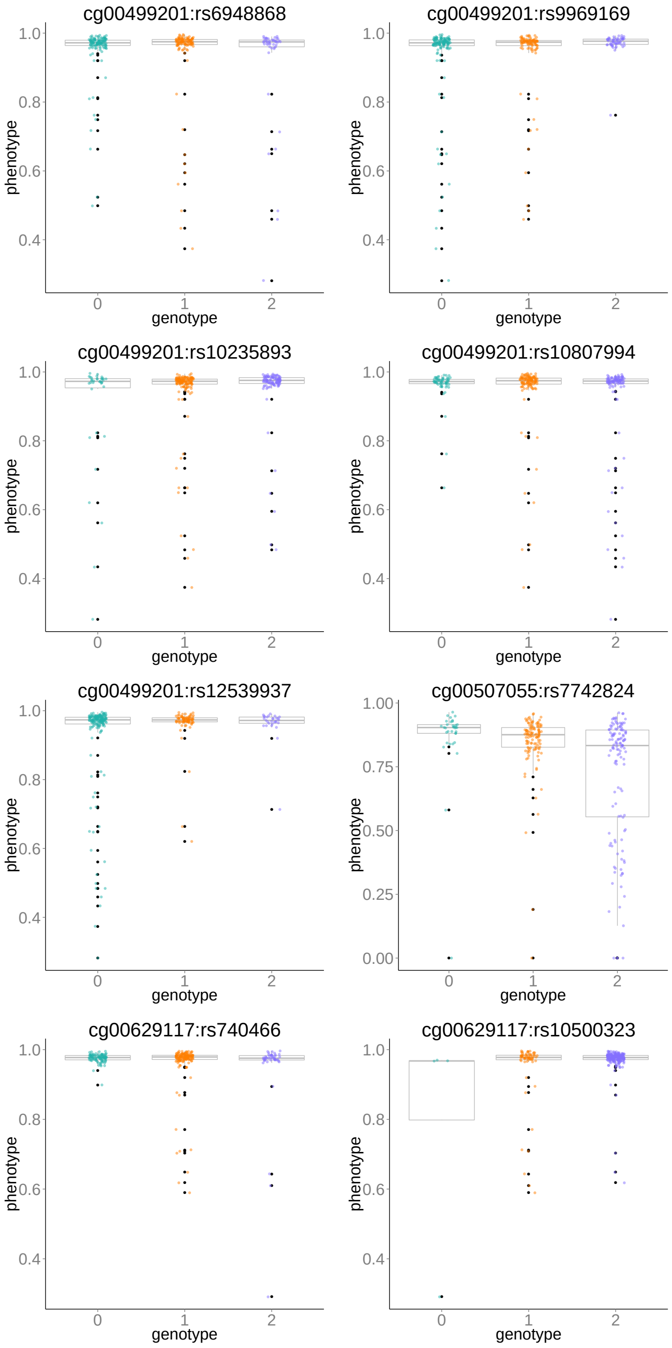

Figure 4: Results CLS from methylation data. From left to right (CpG, SNP) – Panel A: (cg00499201, rs6948868), (cg00499201, rs9969169); Panel B: (cg00499201, rs10235893), (cg00499201, rs10807994); Panel C: (cg00499201, rs12539937), (cg00507055, rs7742824); Panel D: (cg00629117, rs10500323), (cg00629117, rs10500323)

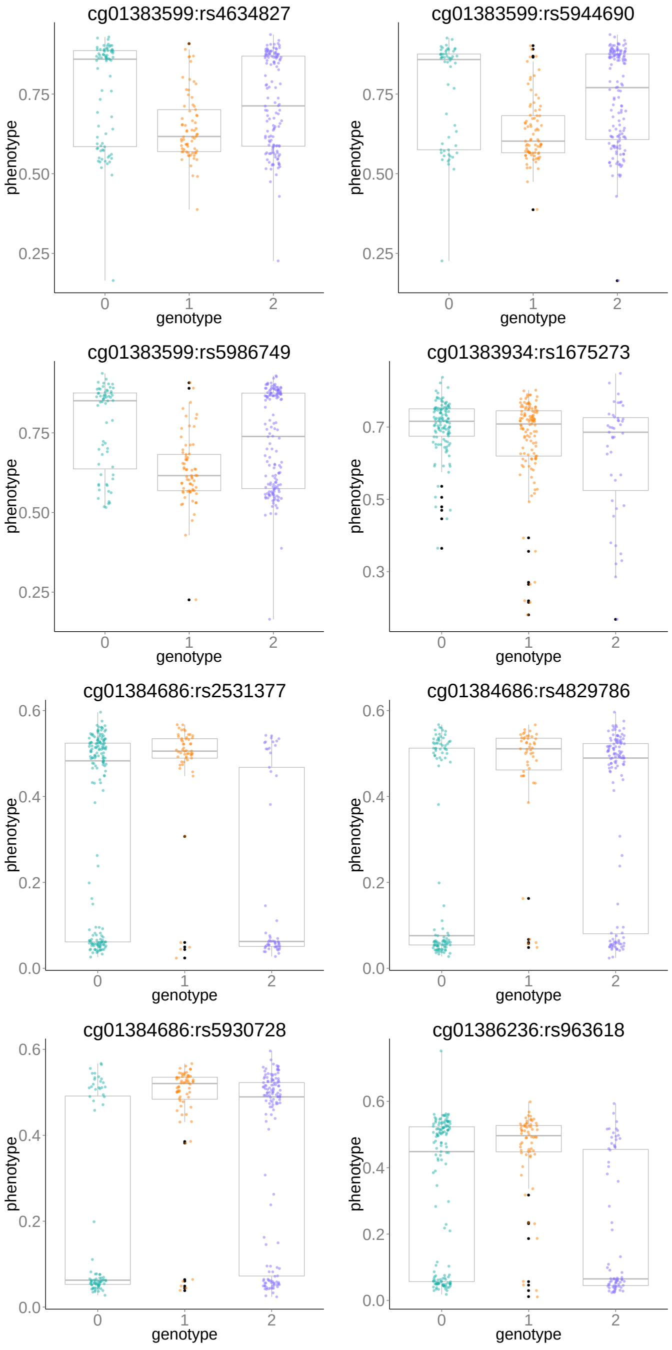

Figure 5: Results Levene from methylation data. From left to right pair (CpG, SNP) – Panel A: (cg01383599, rs4634827), (cg01383599, rs5944690); Panel B: (cg01383599, rs5986749), (cg01383934, rs1675273); Panel C: (cg01384686, rs2531377), (cg01384686, rs4829786); Panel D: (cg01384686, rs5930728), (cg01386236, rs963618)

Distribution Classifier

The correlation matrix corresponding to simulations from the 7 classes of probabilities considered. We notice that the first three classes (the ideal model, the linear variance model, the exponential mean, the bivariate Gaussian) exhibit more similarly among each other, while the exponential residue and the log residue exhibit more statistical similarity with one another than with any distribution.

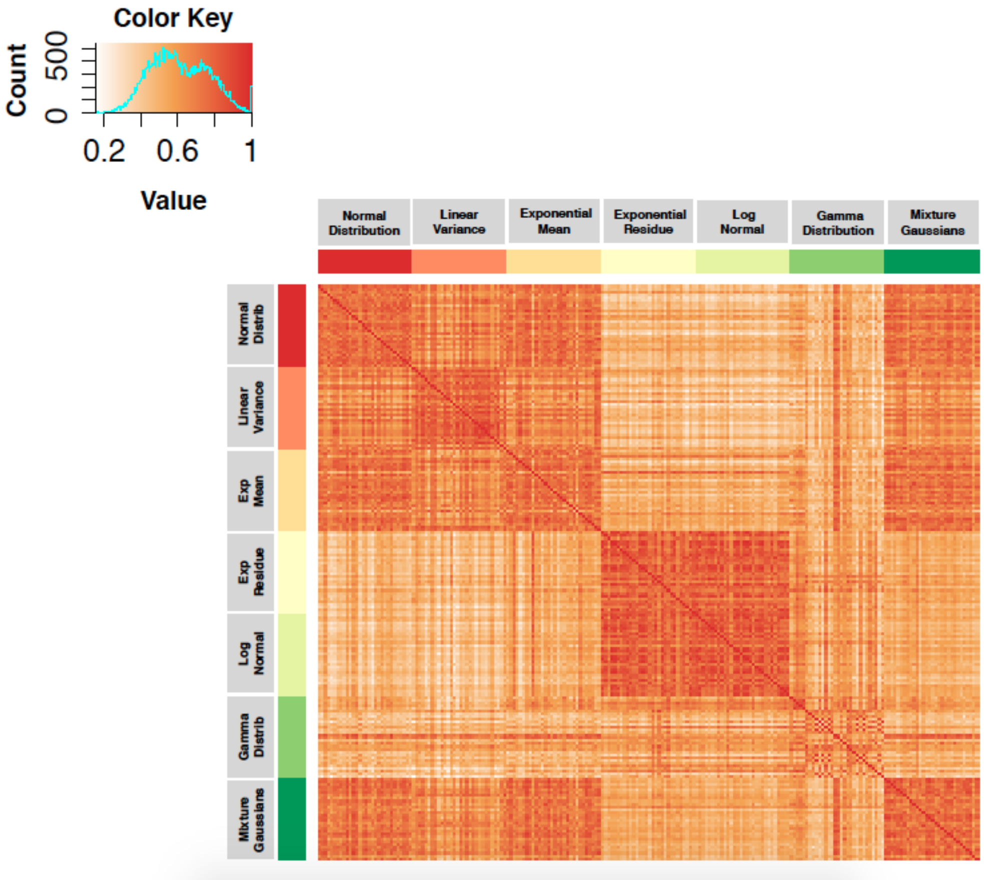

Figure 6: Correlation of Kolmogorov- Smirnov statistical tests for 79 probability distributions across 7 classes of functions

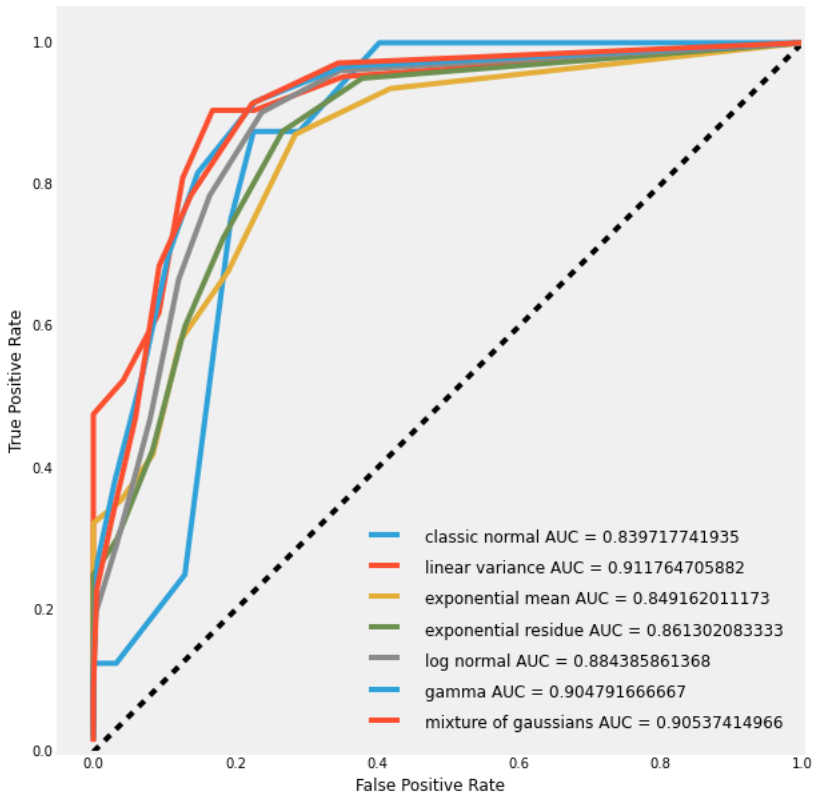

Figure 7: Random Forrest Multi-Class Clasifier, ROC curve, 5-fold validation
