# Supplementary material for: Statistical tests for detecting variance effects in quantitative trait studies
Source: Bioinformatics. 2018 Jul 6;35(2):200–10. doi: 10.1093/bioinformatics/bty565 (PMC6330007; doi:10.1093/bioinformatics/bty565)
Supplement: Supplementary Tables [file bty565_supplementary_tables.pdf]

Supplemental Tables for: Evaluation of statistical tests for detecting variance effects and a Bayesian approach to heteroskedasticity

Bianca Dumitrascu[1], Gregory Darnell[1], Julien Ayroles[1,2] & Barbara E Engelhardt[3,4]

[1] *Lewis-Sigler Institute, Princeton University.*

[2] *Department of Ecology and Evolutionary Biology, Princeton University.*

[3] *Department of Computer Science, Princeton University.*

[4] *Center for Statistics and Machine Learning, Princeton University.*

For methylation data, the significant associations exhibit gamma -like distributions properties when aggregated and, in some cases, they present multi-modal distribution (Mixture of Gaussians or Mixture) behavior within the same genotypic group. More rare labels span additive variance models (AV), mixture of Gaussian models (mixture), log normal models, log normal residue models (LNR), and exponential mean models (Exp. Mean)

Table 1: Variance methylation QTLs discovered by each method. The first row is discoveries for the global FDR (MAF ≥ 0.05). The remaining rows are the findings stratified by mino r allele frequency (MAF Range). All discoveries are made at the greatest FDR at or below 0.0 5.

MAF Range	BTH	DGLM	CLS	Levene
Global	0	3	549	878
0.00-0.05	0	11	4	0
0.05-0.10	0	5	11	5
0.10-0.15	0	1	30	3
0.15-0.20	0	3	85	72
0.20-0.25	0	2	41	213
0.25-0.30	2	4	61	317
0.30-0.35	0	1	70	385
0.35-0.40	0	8	152	464
0.40-0.45	0	3	64	410
0.45-0.50	1	3	81	428

Table 2: Methylation: predicted phenotype distribution labels for a subset of the CLS results

Significant Association		Labels			
CpG region	SNP	Aggregate	Genotype 0	Genotype 1	Genotype 2
cg00149692	rs421473	Gamma	Mixture	LNR	Gamma
cg00259849	rs10103498	Gamma	Gamma	Gamma	Gamma
cg00408876	rs6005232	Gamma	Gamma	Gamma	AV
cg00456685	rs17030583	Gamma	AV	Gamma	Gamma
cg00467278	rs10416031	Gamma	Exp. Mean	AV	Gamma
cg00587301	rs287886	Gamma	Exp. Mean	Gamma	AV
cg00821186	rs6688948	Gamma	Gamma	Gamma	Gamma
cg00972522	rs8045288	Gamma	Gamma	Gamma	Gamma
cg01056775	rs1471921	Gamma	Mixture	AV	Gamma
cg01139696	rs1182188	Gamma	LNR	Gamma	Gamma
cg01199931	rs9976074	Gamma	AV	Gamma	Gamma
cg01365249	rs12146246	Gamma	LNR	Gamma	Gamma
cg01517188	rs7143894	Gamma	Gamma	AV	AV
cg01777786	rs2830742	Gamma	AV	Gamma	Gamma
cg01796338	rs311484	Gamma	Gamma	Gamma	Gamma
cg01987362	rs10512644	Gamma	Gamma	Gamma	Gamma
cg02106043	rs1832739	Gamma	Normal	Gamma	Gamma
cg02394572	rs11769469	Gamma	Gamma	Gamma	Gamma
cg02394572	rs2527686	Gamma	Mixture	Gamma	Gamma
cg02506728	rs2281398	Gamma	Log Normal	Gamma	Gamma
cg02562519	rs7499539	Gamma	Gamma	Gamma	Gamma
cg02709834	rs12723906	Gamma	Gamma	Gamma	LNR
cg02747563	rs6977438	Gamma	AV	Gamma	Gamma
cg02753444	rs1999120	Gamma	Gamma	Gamma	Gamma
cg02775417	rs2301742	Gamma	Gamma	Gamma	Gamma
cg02837488	rs11764572	Gamma	Gamma	Gamma	Gamma
cg03075631	rs4853033	Gamma	Gamma	LNR	-
cg03075631	rs603743	Gamma	Gamma	Gamma	AV
cg03162251	rs7258848	Gamma	Exp. Mean	Gamma	Gamma
cg03227775	rs1182136	Gamma	Gamma	Gamma	Gamma

Table 3: Methylation: predicted phenotype distribution labels for a subset of the Levene results

Significant Association		Labels			
CpG region	SNP	Aggregate	Genotype 0	Genotype 1	Genotype 2
cg00003722	rs416990	Gamma	AV	Gamma	Mixture
cg00077281	rs653765	Gamma	AV	AV	Gamma
cg00259849	rs1036556	Gamma	Gamma	Gamma	Gamma
cg00562665	rs9668031	Gamma	Exp Mean	AV	AV
cg00616572	rs2719701	Gamma	Gamma	Gamma	AV
cg00622384	rs5957080	Gamma	Gamma	Gamma	Gamma
cg00667781	rs6521788	Gamma	Gamma	AV	AV
cg00878023	rs5951592	Gamma	Gamma	AV	Gamma
cg00988678	rs2124399	Gamma	Gamma	Gamma	Gamma
cg01039990	rs6635268	Gamma	Gamma	AV	Gamma
cg01342901	rs2430200	Gamma	Gamma	Gamma	Gamma
cg01370077	rs17318874	Gamma	Gamma	Gamma	Gamma
cg01400468	rs843743	Gamma	Gamma	Gamma	LNR
cg01445689	rs6972776	Gamma	Gamma	Gamma	Gamma
cg01472026	rs7883306	Gamma	Gamma	AV	Gamma
cg01517188	rs7143894	Gamma	Gamma	Gamma	AV
cg01809408	rs12011862	Gamma	Gamma	AV	Exp. Mean
cg01836910	rs2149734	Gamma	AV	Gamma	Gamma
cg01906946	rs6610384	Gamma	Gamma	AV	Gamma
cg02346492	rs12940357	Gamma	Log Normal	Gamma	AV
cg02438576	rs869131	Gamma	Gamma	AV	Gamma
cg02579620	rs5924768	Gamma	Gamma	Gamma	Gamma
cg02609470	rs6773566	Gamma	Normal	AV	LNR
cg02693068	rs5963731	Gamma	Gamma	AV	Gamma
cg02852025	rs3735275	AV	AV	AV	Gamma
cg02864732	rs209217	Gamma	AV	AV	Gamma
cg02931660	rs5963731	Gamma	Gamma	LNR	Gamma
cg03028851	rs2266888	Gamma	Gamma	Exp Mean	Gamma
cg03146649	rs4784754	Gamma	Gamma	Gamma	LNR
cg03191359	rs5918494	Gamma	Gamma	Gamma	Gamma

Table 4: Number of genotype-expression variance associations discovered by each method. T he first row is discoveries for the global FDR (MAF ≥ 0.05). The remaining rows are the findings stratified by minor allele frequency (MAF Range). All discoveries are made at the g reatest FDR at or below 0.05.

MAF Range	BTH	DGLM	CLS
Global	0	1	0
0.00-0.05	0	0	0
0.05-0.10	0	0	0
0.10-0.15	0	0	0
0.15-0.20	0	0	0
0.20-0.25	0	0	0
0.25-0.30	0	0	0
0.30-0.35	6	0	0
0.35-0.40	0	0	0
0.40-0.45	0	0	0
0.45-0.50	0	0	0

Table 5: Number of genotype-covariate variance associations discovered by each method. All discoveries are made at the greatest FDR at or below 0.05.

Covariate	BTH	DGLM	CLS
Age	0	5	15
BMI	0	1	0
Sex	4	0	2
Smoke	0	0	0

Table 6: CAP Covariates: list of all results

Gene	Covariate	Method
C14orf166	sex	BTH
DC2	sex	BTH
FRG1	sex	BTH
TMEM14B	sex	BTH
ATOX1	age	*dglm*
B3GNTL1	age	*dglm*
CYorf15B	bmi	*dglm*
LOC124220	age	*dglm*
PLEKHF1	age	*dglm*
PYGO2	age	*dglm*
AP1S2	age	CLS
APOC1	age	CLS
ARID5B	age	CLS
C11orf74	age	CLS
CRY1	age	CLS
CYorf15A	sex	CLS
DCUN1D3	age	CLS
EIF2C2	age	CLS
FERMT2	age	CLS
GALC	age	CLS
GTSF1	age	CLS
IFNAR2	age	CLS
LOC440157	age	CLS
MAP3K4	age	CLS
RALGPS2	age	CLS
SLC29A3	age	CLS
SLC35A4	age	CLS
