# Supplementary material for: Statistical tests for detecting variance effects in quantitative trait studies
Source: Bioinformatics. 2018 Jul 6;35(2):200–10. doi: 10.1093/bioinformatics/bty565 (PMC6330007; doi:10.1093/bioinformatics/bty565)
Supplement: Supplementary Text [file bty565_supplementary_text.pdf]

Supplemental text for: Evaluation of statistical tests for detecting variance effects and a Bayesian approach to heteroskedasticity

Bianca Dumitrascu[1], Gregory Darnell[1], Julien Ayroles[1,2] & Barbara E Engelhardt[3,4]

[1]*Lewis-Sigler Institute, Princeton University.*

[2]*Department of Ecology and Evolutionary Biology, Princeton University.*

[3]*Department of Computer Science, Princeton University.*

[4]*Center for Statistics and Machine Learning, Princeton University.*

1 Model information

In this subsection we describe the calculation of the Bayes Factors in detail. Specifically, we show how to perform posterior calculations, estimation of parameters, and multivariate Laplace approximation. We further compute the derivatives necessary to perform a multivariate Laplace approximation,and show how different priors for the α can be incorporated into this computation. We exemplify with Laplace and Cauchy (used in BTH) priors.

In the following we present the computations integrated into our BTH implementation. Recall that the quantity of interest is the Bayes Factor

$$BF = \frac{\Pr(\mathbf{y}|\mathbf{x}, \mathbf{H_1})}{\Pr(\mathbf{y}|\mathbf{x}, \mathbf{H_0})} = \frac{\int_0^\infty \int_0^\infty \int_0^\infty \Pr(\mathbf{y}|\mathbf{x}; \beta_\mathbf{0}, \sigma^\mathbf{2}, \alpha) \Pr(\beta_\mathbf{0}, \sigma^\mathbf{2}) \Pr(\alpha) \mathbf{d}\beta_\mathbf{0} \mathbf{d}\sigma^\mathbf{2} \mathbf{d}\alpha}{\int_0^\infty \int_0^\infty \Pr(\mathbf{y}|\mathbf{x}; \beta_\mathbf{0}, \sigma^\mathbf{2}, \alpha = \mathbf{1}) \Pr(\beta_\mathbf{0}, \sigma^\mathbf{2}) \Pr(\alpha) \mathbf{d}\beta_\mathbf{0} \mathbf{d}\sigma^\mathbf{2}}. \quad (1)$$

We will make use of known fact that for any β the following equality holds:

$$\Pr(\mathbf{y}\big|\mathbf{x};\beta_\mathbf{0},\sigma^\mathbf{2},\alpha) = \frac{\Pr(\mathbf{y}\big|\mathbf{x};\beta_\mathbf{0},\sigma^\mathbf{2},\alpha,\beta)\Pr(\beta\big|\sigma^\mathbf{2})}{\Pr(\beta\big|\mathbf{y},\mathbf{x};\beta_\mathbf{0},\sigma^\mathbf{2},\alpha,\beta)} \tag{2}$$

To enable an easier computation and implementation, we use the following notation:

$$S_0 = \sum \alpha^{x_i} \quad S_1 = \sum x_i \cdot \alpha^{x_i} \quad S_2 = \sum x_i^2 \cdot \alpha^{x_i} \quad S_3 = \sum x_i^3 \cdot \alpha^{x_i} \quad S_4 = \sum x_i^4 \cdot \alpha^{x_i}$$

$$R_1 = \sum y_i \cdot \alpha^{x_i} \quad R_2 = \sum y_i^2 \cdot \alpha^{x_i} \quad R_3 = \sum y_i^3 \cdot \alpha^{x_i}$$

$$Q1 = \sum y_i x_i \cdot \alpha^{x_i} \quad Q2 = \sum y_i x_i^2 \cdot \alpha^{x_i} \quad Q3 = \sum y_i x_i^3 \cdot \alpha^{x_i}$$

$$O1 = \sum y_i^2 x_i \cdot \alpha^{x_i} \quad O2 = \sum y_i^2 x_i^2 \cdot \alpha^{x_i} \quad O3 = \sum y_i^2 x_i^3 \cdot \alpha^{x_i}$$

$$G = \sigma^2 \cdot v + S_2$$

Let $\mathcal{W}$ be the heteroskedastic variance matrix

$$\mathcal{W} = \sigma^2 \cdot \begin{pmatrix} \alpha^{-x_1} & 0 & \cdots & 0 \\ 0 & \alpha^{-x_2} & \cdots & 0 \\ \vdots & \vdots & \ddots & \vdots \\ 0 & 0 & \cdots & \alpha^{-x_n} \end{pmatrix},$$

We compute the likelihood term $\Pr(\mathbf{y}\big|\mathbf{x};\beta_\mathbf{0},\sigma^\mathbf{2},\alpha)$, noting that its distribution is normal distribution, $\mathbf{y}\big|\mathbf{x};\beta_\mathbf{0},\sigma^\mathbf{2},\alpha,\beta \sim \mathcal{N}(\beta\mathbf{x}+\beta_\mathbf{0}\mathbf{1},\mathcal{W})$,

$$\Pr(\mathbf{y}\big|\mathbf{x};\beta_\mathbf{0},\sigma^\mathbf{2},\alpha,\beta) = \prod_\mathbf{i}^\mathbf{n} \mathcal{N}(\beta\mathbf{x_i}+\beta_\mathbf{0},\sigma^\mathbf{2}\alpha^{-\mathbf{x_i}}) = \prod_\mathbf{i}^\mathbf{n} \frac{\mathbf{1}}{\sqrt{\mathbf{2}\sigma^\mathbf{2}\cdot\alpha^{-\mathbf{x_i}}}} \exp\left\{\frac{(\mathbf{y}-\beta_\mathbf{0}-\beta\mathbf{x_i})^\mathbf{2}}{\mathbf{2}\sigma^\mathbf{2}\cdot\alpha^{-\mathbf{x_i}}}\right\}$$

$$= (2\pi)^{-\frac{n}{2}} \cdot \sigma^{2\frac{-n}{2}} \cdot \alpha^{\frac{\sum x_i}{2}} \cdot \exp\left\{-\frac{1}{2\sigma^2}(R_2+\beta_0^2\cdot S_0+\beta^2\cdot S_2+2\cdot\beta_0\beta S_1-2\beta Q_1-2\beta_0 R_1)\right\},$$

and the posterior term $\Pr(\beta|y,x,\sigma^2,\alpha,\beta_0)$, noting that $\beta|y,x,\sigma^2,\alpha,\beta_0 \sim \mathcal{N}(\hat{\beta},V_\beta)$, where

$$V_\beta = (v + x^T\cdot\mathcal{W}^{-1}\cdot x)^{-1}$$

$$\hat{\beta} = V_\beta \cdot x^T \cdot \mathcal{W}^{-1}\cdot(y-\beta_0)$$

. Therefore,

$$\Pr(\beta|y,x,\sigma^2,\alpha,\beta_0) = (2\pi)^{-\frac{1}{2}} \cdot \left(\frac{\sigma^2}{G}\right)^{-\frac{1}{2}} \exp\left\{-\frac{\left(\beta - \frac{Q_1-\beta_0\cdot S_1}{G}\right)^2}{\frac{2\sigma^2}{G}}\right\} =$$

$$= (2\pi)^{-\frac{1}{2}} \cdot \left(\frac{\sigma^2}{G}\right)^{-\frac{1}{2}} \cdot$$

$$\exp\left\{-\left(\beta^2 \cdot \frac{G}{2\sigma^2} - \beta \cdot \frac{Q_1}{\sigma^2} + \beta \cdot \beta_0 \cdot \frac{S_1}{\sigma^2} + \beta_0^2 \cdot \frac{S_1^2}{2\sigma^2 \cdot (G)} - \beta_0 \cdot \frac{Q1 \cdot S_1}{G} + \frac{Q_1^2}{2\sigma^2 \cdot (G)}\right)\right\}$$

Finally, plugging the previous results into Equation (2), we obtain:

$$\begin{aligned}\Pr(\mathbf{y}|\mathbf{x};\boldsymbol{\beta_0},\boldsymbol{\sigma^2},\alpha) &= (2\pi)^{-\frac{n}{2}+\frac{1}{2}} \cdot \sigma^{2(-\frac{n}{2}+\frac{1}{2})} \cdot \alpha^{\frac{1}{2}\cdot\sum_{i=1}^n x_i} \cdot v^{\frac{1}{2}} \cdot (G)^{-\frac{1}{2}} \\ &\cdot \exp\left\{-\frac{1}{2\sigma^2}\left[R_2 - \frac{Q_1^2}{G} - 2\beta_0 \cdot \left(R_1 - \frac{Q_1 \cdot S_1}{G}\right) + \beta_0^2\left(S_0 - \frac{S_1^2}{G}\right)\right]\right\} \\ &= K \cdot \sigma^{2(-\frac{n}{2}+\frac{1}{2})} \cdot \alpha^{\frac{1}{2}\cdot\sum_{i=1}^n x_i} \cdot (G)^{-\frac{1}{2}} \\ &\cdot \exp\left\{-\frac{1}{2\sigma^2}\left[R_2 - \frac{Q_1^2}{G} - 2\beta_0 \cdot \left(R_1 - \frac{Q_1 \cdot S_1}{G}\right) + \beta_0^2\left(S_0 - \frac{S_1^2}{G}\right)\right]\right\}.\end{aligned} \tag{3}$$

Notice that all Qs, Rs, Ss are functions of α.

Equations (2) and (3), together with the appropriate prior probabilities, are used to approximate the Bayes factors using a multivariate Laplace approximation. In particular, both the numerator and the denominator can be written as $\int e^{-nh(x)}\text{dx} = e^{-nh(\hat{x})} \cdot (2\pi)^{\frac{d}{2}} \cdot |\Sigma^{-1}|^{\frac{1}{2}} \cdot n^{-\frac{d}{2}}$, where $\hat{x} = argmin_x h(x)$, and where $|\Sigma^{-1}|$ is the determinant of the Jacobian of h evaluated at $\hat{x}$. For a more general case we consider a Laplace prior on $\log(\alpha)$ which requires the spitting of the integral domain at 0. Similar calculations follow in the case of a Cauchy prior, in which the splitting of the domain is unnecessary.

In detail, we look for functions h_{1a}, h_{1b}, and h_2 corresponding to the different parts of the Bayes Factor: numerator (h_{1a}, h_{1b}), and to the denominator (h_{1a}). Consequently, we can write the

numerator as a sum of 2 3-dimensional integrals, and the denominator as a 2 dimensional integral, such that:

$$BF = \frac{\int_0^1 \int\int e^{-n\cdot h_{1a}(\beta_0,\sigma^2,\alpha)} d\beta_0 d\sigma^2 d\alpha + \int_1^\infty \int\int e^{-n\cdot h_{1b}(\beta_0,\sigma^2,\alpha)} d\beta_0 d\sigma^2 d\alpha}{\int\int e^{-n\cdot h_2(\beta_0,\sigma^2,\alpha=1)} d\beta_0 d\sigma^2} \tag{4}$$

where

$$\begin{aligned} -n\cdot h_{1a}(\beta_0,\sigma^2,\alpha) &= \log\left(\Pr(y|x;\beta_0,\sigma^2,\alpha)\cdot\Pr(\sigma^2,\beta_0)\cdot\Pr_{<1}(\alpha)\right) \\ -n\cdot h_{1b}(\beta_0,\sigma^2,\alpha) &= \log\left(\Pr(y|x;\beta_0,\sigma^2,\alpha)\cdot\Pr(\sigma^2,\beta_0)\cdot\Pr_{>1}(\alpha)\right) \\ -n\cdot h_2(\beta_0,\sigma^2) &= \log\left(\Pr(y|x;\beta_0,\sigma^2,\alpha=1)\cdot\Pr(\sigma^2,\beta_0)\right). \end{aligned} \tag{5}$$

Using the multivariate Laplace approximation with $d = 3$ and $d = 2$, the Bayes factor (BF) can be written as

$$n^{-\frac{1}{2}}\cdot\frac{e^{-nh_{1a}(\hat{\beta_0}(1a),\hat{\Sigma^2}(1a),\hat{\alpha}(1a))}\cdot(2\pi)^{\frac{3}{2}}\cdot|\Sigma^2_{1a}|^{\frac{1}{2}} + e^{-nh_{1b}(\hat{\beta_0}(1b),\hat{\Sigma^2}(1b),\hat{\alpha}(1b))}\cdot(2\pi)^{\frac{3}{2}}\cdot|\Sigma^2_{1b}|^{\frac{1}{2}}}{e^{-nh_2(\hat{\beta_0}(2),\hat{\sigma^2}(2),\hat{\alpha}(2))}\cdot(2\pi)^{\frac{2}{2}}\cdot|\Sigma^2_2|^{\frac{1}{2}}}, \tag{6}$$

or simpler, $\log BF = \log T_1 + \log T_2$, where

$$\begin{aligned} \log T_1 &= -n\cdot(h_{1a}() - h_2()) + \frac{1}{2}\cdot\log(2\pi) + \frac{1}{2}\left(\log\sigma^2_{1a} - \log\sigma^2_2\right) - \frac{1}{2}\cdot\log n \\ \log T_2 &= -n\cdot(h_{1b}() - h_2()) + \frac{1}{2}\cdot\log(2\pi) + \frac{1}{2}\left(\log\sigma^2_{1b} - \log\sigma^2_2\right) - \frac{1}{2}\cdot\log n. \end{aligned} \tag{7}$$

Compute the terms associated with h_{1a}

$$
\begin{aligned}
&h_{1a}(\beta_0, \sigma^2, \alpha) = \\
&= -\frac{1}{n} \log \Bigg\{ K \cdot \sigma^{2\cdot(-\frac{n}{2}+\frac{1}{2})} \cdot \alpha^{\frac{1}{2}\cdot\sum_{i=1}^{n} x_i} \cdot G^{-\frac{1}{2}} \\
&\cdot \exp\Bigg\{ -\frac{1}{2\sigma^2}\left[R_2 - \frac{Q_1^2}{G} - 2\beta_0 \cdot \left(R_1 - \frac{Q_1 \cdot S_1}{G}\right) + \beta_0^2\left(S_0 - \frac{S_1^2}{G}\right)\right]\Bigg\} \\
&\cdot \frac{\theta_2^{\theta_1}}{\Gamma(\theta_1)} \sigma^{2\cdot(-\theta_1-1)} \exp\left(-\frac{\theta_2}{\sigma^2}\right) \cdot \frac{1}{2b} \cdot \alpha^{-1+\frac{1}{b}} \Bigg\} = \\
&= -\frac{1}{n}\Bigg\{ \log K' + \left(-\frac{n}{2}+\frac{1}{2}\right)\log\sigma^2 + \frac{\log\alpha}{2}\sum_{i=1}^{n} x_i - \frac{1}{2}\log(G) \\
&-\frac{1}{2\sigma^2}\cdot\left[R_2 - \frac{Q_1^2}{G} - 2\beta_0 \cdot \left(R_1 - \frac{Q_1 \cdot S_1}{G}\right) + \beta_0^2\left(S_0 - \frac{S_1^2}{G}\right)\right] + \\
&(-\theta_1 - 1)\log\sigma^2 - \frac{\theta_2}{\sigma^2} - \log(2b) + \left(\frac{1}{b} - 1\right)\log\alpha \Bigg\}
\end{aligned}
\tag{8}
$$

As the constant K' is common to all the terms involved in the computation, let the function h_{1a} be $-\frac{1}{n}(L_0 + L_1 + L_2 + L_3 + Prior_{\sigma^2} + Prior_\alpha)$, with

$$
\begin{aligned}
L_0 &= \left(-\frac{n}{2}+\frac{1}{2}\right)\log\sigma^2 + \frac{\log\alpha}{2}\sum_{i=1}^{n} x_i - \frac{1}{2}\log(G) \\
L_1 &= -\frac{1}{2\sigma^2}\cdot\left(R_2 - \frac{Q_1^2}{G}\right) \\
L_2 &= \frac{1}{\sigma^2}\cdot\beta_0\cdot\left(R_1 - \frac{Q_1 \cdot S_1}{G}\right) \\
L_3 &= -\frac{1}{2\sigma^2}\cdot\beta_0^2\left(S_0 - \frac{S_1^2}{G}\right) \\
Prior_{\sigma^2} &= (-\theta_1 - 1)\log\sigma^2 - \frac{\theta_2}{\sigma^2} \\
Prior_\alpha &= -\log(2b) + \left(\frac{1}{b} - 1\right)\log\alpha
\end{aligned}
\tag{9}
$$

. For each term we compute single and double derivatives with respect to α, σ^2, β_0.

Compute the derivatives of L_0

$$L_0 = \left(-\frac{n}{2} + \frac{1}{2}\right) \log \sigma^2 + \frac{\log \alpha}{2} \sum_{i=1}^{n} x_i - \frac{1}{2} \log(G) \tag{10}$$

The following terms which contain L_0 are needed:

$$\begin{aligned}
\frac{\partial}{\partial \alpha} L_0 &= \frac{1}{2\alpha} \cdot \left(\sum_{i=1}^{n} x_i - \frac{S_3}{G} \right) \\
\frac{\partial}{\partial \beta_0} L_0 &= 0 \\
\frac{\partial}{\partial \sigma^2} L_0 &= \left(-\frac{n}{2} + \frac{1}{2}\right) \cdot \frac{1}{\sigma^2} - \frac{1}{2} \cdot \frac{v}{G} \\
\frac{\partial^2}{\partial \alpha^2} L_0 &= -\frac{1}{2\alpha^2} \cdot \left(\sum_{i=1}^{n} x_i - \frac{S_3}{G} \right) + \frac{1}{2\alpha} \left(-\frac{1}{\alpha} \cdot \frac{S_4}{G} + \frac{1}{\alpha} \cdot \frac{S_3^2}{(G)^2} \right) \\
&= -\frac{1}{2\alpha^2} \cdot \left(\sum_{i=1}^{n} x_i - \frac{S_3}{G} \right) + \frac{1}{2\alpha^2} \left(-\frac{S_4}{G} + \frac{S_3^2}{(G)^2} \right) \\
&= \frac{1}{2\alpha^2} \cdot \frac{1}{G}(S_3 - S_4) + \frac{1}{2\alpha^2} \cdot \frac{S_3^2}{G^2} - \frac{1}{2\alpha^2} \sum_{i=1}^{n} x_i \qquad (11) \\
\frac{\partial^2}{\partial \beta_0^2} L_0 &= 0 \\
\frac{\partial^2}{\partial (\sigma^2)^2} L_0 &= -\left(-\frac{n}{2} + \frac{1}{2} \right) \cdot \frac{1}{(\sigma^2)^2} + \frac{1}{2} \cdot \frac{v^2}{(G)^2} \\
\frac{\partial^2}{\partial \beta_0 \partial \alpha} L_0 &= 0 \\
\frac{\partial^2}{\partial \sigma^2 \partial \alpha} L_0 &= \frac{1}{2\alpha} \cdot \frac{v \cdot S_3}{(G)^2} \\
\frac{\partial^2}{\partial \beta_0 \partial \sigma^2} L_0 &= 0
\end{aligned}$$

Compute the derivatives of L_1

$$L_1 = -\frac{1}{2\sigma^2} \cdot \left(R_2 - \frac{Q_1^2}{G} \right) \tag{12}$$

The following terms containing L_1 are needed:

$$\begin{aligned}
\frac{\partial}{\partial \alpha} L_1 &= -\frac{1}{2\sigma^2} \cdot \left(\frac{O_1}{\alpha} - \frac{2 \cdot Q_1 Q_2}{\alpha} \cdot \frac{1}{G} + \frac{Q_1^2 \cdot S_3}{\alpha \cdot (G)^2} \right) \\
&= -\frac{1}{2\sigma^2 \alpha} \cdot \left(O_1 - \frac{2 \cdot Q_1 Q_2}{G} + \frac{Q_1^2 \cdot S_3}{(G)^2} \right) \\
\frac{\partial}{\partial \beta} L_1 &= 0 \\
\frac{\partial}{\partial \sigma^2} L_1 &= \frac{1}{2(\sigma^2)^2} \left(R_2 - \frac{Q_1^2}{G} \right) - \frac{1}{2\sigma^2} \cdot \frac{Q_1^2 v}{(G)^2} \\
\frac{\partial^2}{\partial \alpha^2} L_1 &= \frac{1}{2\sigma^2 \alpha^2} \cdot \left(O_1 - \frac{2 \cdot Q_1 Q_2}{G} + \frac{Q_1^2 \cdot S_3}{(G)^2} \right) - \frac{1}{2\sigma^2 \alpha^2} \cdot \\
&\left(O_2 - \frac{2Q_2^2 + 2Q_1 Q_3}{G} + \frac{4Q_1 Q_2 \cdot S_3 + Q_1^2 \cdot S_4}{G^2} - \frac{2 \cdot Q_1^2 \cdot S_3^2}{G^3} \right) \\
\frac{\partial^2}{\partial \beta_0^2} L_1 &= 0 \\
\frac{\partial^2}{\partial (\sigma^2)^2} L_1 &= -\frac{1}{(\sigma^2)^3} \left(R_2 - \frac{Q_1^2}{G} \right) + \frac{1}{(\sigma^2)^2} \cdot \frac{Q_1^2 \cdot v}{G^2} + \frac{1}{\sigma^2} \cdot \frac{Q_1^2 \cdot v^2}{G^3} \\
\frac{\partial^2}{\partial \beta_0 \partial \alpha} L_1 &= 0 \\
\frac{\partial^2}{\partial \sigma^2 \partial \alpha} L_1 &= \frac{1}{2(\sigma^2)^2} \cdot \frac{1}{\alpha} \left(O_1 - \frac{2Q_1 Q_2}{G} + \frac{Q_1^2 \cdot S_3}{G^2} \right) \\
&- \frac{1}{2\sigma^2} \cdot \frac{1}{\alpha} \left(\frac{2Q_1 \cdot Q_2 \cdot v}{G^2} - \frac{2 \cdot Q_1^2 \cdot S_3 \cdot v}{G^3} \right) \\
\frac{\partial^2}{\partial \beta_0 \partial \sigma^2} L_1 &= 0
\end{aligned} \tag{13}$$

Compute the derivatives of L_2

$$L_2 = \frac{1}{\sigma^2} \cdot \beta_0 \cdot \left(R_1 - \frac{Q_1 \cdot S_1}{G} \right) \tag{14}$$

The following terms containing L_2 are needed:

$$\begin{aligned}
\frac{\partial}{\partial \alpha} L_2 &= \frac{\beta_0}{\sigma^2 \cdot \alpha} \cdot \left(Q_1 - \frac{Q_2 \cdot S_1}{G} - \frac{Q_1 \cdot S_2}{G} + \frac{Q_1 \cdot S_1 \cdot S_3}{G^2} \right) \\
\frac{\partial}{\partial \beta} L_2 &= \frac{1}{\sigma^2} \left(R_1 - \frac{Q_1 \cdot S_1}{G} \right)
\end{aligned} \tag{15}$$

$$
\begin{aligned}
\frac{\partial}{\partial \sigma^2} L_2 &= -\frac{\beta_0}{(\sigma^2)^2}\left(R_1 - \frac{Q_1 \cdot S_1}{G}\right) + \frac{\beta_0}{\sigma^2} \cdot \frac{Q_1 \cdot S_1 \cdot v}{G^2} \\
\frac{\partial^2}{\partial \alpha^2} L_2 &= -\frac{\beta_0}{\sigma^2}\frac{1}{\alpha^2}\left(Q_1 - \frac{Q_2 \cdot S_1 + Q_1 \cdot S_2}{G} + \frac{Q_1 \cdot S_1 \cdot S_3}{G^2}\right) + \\
&\quad + \frac{\beta_0}{\sigma^2} \cdot \frac{1}{\alpha^2}\Bigg(Q_2 - \frac{Q_3 \cdot S_1 + Q_2 \cdot S_2 + Q_2 \cdot S_2 + Q_1 \cdot S_3}{G} + \frac{S_3 \cdot (Q_2 \cdot S_1 + Q_1 \cdot S_2)}{G^2} + \\
&\quad + \frac{Q_2 \cdot S_1 \cdot S_3 + Q_1 \cdot S_2 \cdot S_3 + Q_1 \cdot S_1 \cdot S_4}{G^2} - \frac{2 \cdot S_3 \cdot Q_1 \cdot S_1 \cdot S_3}{G^3}\Bigg) \\
\frac{\partial^2}{\partial \beta_0^2} L_2 &= 0 \\
\frac{\partial^2}{\partial (\sigma^2)^2} L_2 &= \frac{2}{(\sigma^2)^3} \cdot \beta_0\left(R_1 - \frac{Q_1 \cdot S_1}{G}\right) - \frac{\beta_0}{(\sigma^2)^2} \cdot \frac{Q_1 \cdot S_1 \cdot v}{G^2} + \\
&\quad + \left(-\frac{1}{(\sigma^2)^2}\right) \cdot \beta_0 \cdot \frac{Q_1 \cdot S_1 \cdot v}{G^2} - \frac{2}{\sigma^2} \cdot \beta_0 \cdot \frac{Q_1 \cdot S_1 \cdot v^2}{G^3} \\
\frac{\partial^2}{\partial \beta_0 \partial \alpha} L_2 &= \frac{1}{\sigma^2} \cdot \frac{1}{\alpha}\left(Q_1 - \frac{Q_2 \cdot S_1}{G} - \frac{Q_1 \cdot S_2}{G} + \frac{Q_1 \cdot S_1 \cdot S_3}{G^2}\right) \\
\frac{\partial^2}{\partial \sigma^2 \partial \alpha} L_2 &= -\beta_0 \cdot \frac{1}{(\sigma^2)^2} \cdot \frac{1}{\alpha}\left(Q_1 - \frac{Q_2 \cdot S_1}{G} - \frac{Q_1 \cdot S_2}{G} + \frac{Q_1 \cdot S_1 \cdot S_3}{G^2}\right) + \\
&\quad + \beta_0 \cdot \frac{1}{\sigma^2} \cdot \frac{1}{\alpha}\left(\frac{v \cdot (Q_2 \cdot S_1 + Q_1 \cdot S_2)}{G^2} - 2 \cdot \frac{Q_1 \cdot S_1 \cdot S_3 \cdot v}{G^3}\right) \\
\frac{\partial^2}{\partial \beta_0 \partial \sigma^2} L_2 &= -\frac{1}{(\sigma^2)^2}\left(R_1 - \frac{Q_1 \cdot S_1}{G}\right) + \frac{1}{\sigma^2} \cdot \frac{Q_1 \cdot S_1 \cdot v}{G^2}
\end{aligned}
\tag{16}
$$

Compute derivative of L_3

$$
L_3 = \frac{1}{2\sigma^2} \cdot \beta_0^2 \cdot \left(S_0 - \frac{S_1^2}{G}\right) \tag{17}
$$

The following terms which contain L_3 are needed:

$$
\begin{aligned}
\frac{\partial}{\partial\alpha}L_3 &= -\frac{1}{2\sigma^2}\cdot\beta_0^2\cdot\frac{1}{\alpha}\left(S_1-\frac{2\cdot S_1\cdot S_2}{G}+\frac{S_1^2\cdot S_3}{G^2}\right)\\
\frac{\partial}{\partial\beta}L_3 &= -\frac{\beta_0}{\sigma^2}\left(S_0-\frac{S_1^2}{G}\right)\\
\frac{\partial}{\partial\sigma^2}L_3 &= \frac{\beta_0^2}{2(\sigma^2)^2}\left(S_0-\frac{S_1^2}{G}\right)-\frac{1}{2\sigma^2}\cdot\beta_0^2\cdot\frac{S_1^2\cdot v}{G^2}
\end{aligned}
\tag{18}
$$

$$
\begin{aligned}
\frac{\partial^2}{\partial\alpha^2}L_3 &= \frac{1}{2\sigma^2}\cdot\frac{\beta_0^2}{\alpha^2}\left(S_1-\frac{2\cdot S_1\cdot S_2}{G}+\frac{S_1^2\cdot S_3}{G^2}\right)-\\
&-\frac{1}{2\sigma^2}\cdot\frac{\beta_0^2}{\alpha^2}\left(S_2-\frac{2(S_2^2+S_1\cdot S_3)}{G}+\frac{2\cdot S_1\cdot S_2\cdot S_3}{G^2}+\frac{2\cdot S_1\cdot S_2\cdot S_3+S_1^2\cdot S_4}{G^2}-\frac{2\cdot S_1^2\cdot S_3^2}{G^3}\right)\\
\frac{\partial^2}{\partial\beta_0^2}L_3 &= -\frac{1}{\sigma^2}\left(S_0-\frac{S_1^2}{G}\right)\\
\frac{\partial^2}{\partial(\sigma^2)^2}L_3 &= -\frac{1}{(\sigma^2)^3}\cdot\beta_0^2\left(S_0-\frac{S_1^2}{G}\right)+\frac{1}{(\sigma^2)^2}\cdot\beta_0^2\cdot\frac{S_1^2\cdot v}{G^2}+\frac{1}{\sigma^2}\cdot\beta_0^2\cdot\frac{S_1^2\cdot v^2}{G^3}\\
\frac{\partial^2}{\partial\beta_0\partial\alpha}L_3 &= -\frac{1}{\sigma^2}\cdot\beta_0\cdot\frac{1}{\alpha}\left(S_1-\frac{2\cdot S_1\cdot S_2}{G}+\frac{S_1^2\cdot S_3}{G^2}\right)\\
\frac{\partial^2}{\partial\sigma^2\partial\alpha}L_3 &= +\frac{1}{2(\sigma^2)^2}\cdot\beta_0^2\cdot\frac{1}{\alpha}\left(S_1-\frac{2\cdot S_1\cdot S_2}{G}+\frac{S_1^2\cdot S_3}{G^2}\right)-\\
&-\frac{1}{2\sigma^2}\cdot\beta_0^2\cdot\frac{1}{\alpha}\left(+\frac{2\cdot S_1\cdot S_2\cdot v}{G^2}-\frac{2\cdot S_1^2\cdot S_3\cdot v}{G^3}\right)\\
\frac{\partial^2}{\partial\beta_0\partial\sigma^2}L_3 &= \frac{\beta_0}{(\sigma^2)^2}\left(S_0-\frac{S_1^2}{G}\right)-\frac{\beta_0}{\sigma^2}\cdot\frac{S_1^2\cdot v}{G^2}
\end{aligned}
\tag{19}
$$

Compute the derivatives of $Prior_{\sigma^2}$ Due to the form of the prior $Prior_{\sigma^2}=(-\theta_1-1)\log\sigma^2-\frac{\theta_2}{\sigma^2}$ the only non-zero partial derivatives are:

$$
\begin{aligned}
\frac{\partial}{\partial\sigma^2}Prior_{\sigma^2} &= (-1-\theta_1)\cdot\frac{1}{\sigma^2}+\frac{\theta_2}{(\sigma^2)^2}\\
\frac{\partial^2}{\partial(\sigma^2)^2}Prior_{\sigma^2} &= (1+\theta_1)\cdot\frac{1}{(\sigma^2)^2}-\frac{2\cdot\theta_2}{(\sigma^2)^3}
\end{aligned}
\tag{20}
$$

Compute the derivatives of $Prior_{\alpha,a}$ - which correspond to h_{1a} Due to the form of the prior $Prior_{\alpha} = \log\left(\frac{1}{2b}\right) + \left(\frac{1}{b} - 1\right)\log\alpha$ the only non-zero partial derivatives are:

$$\begin{aligned}\frac{\partial}{\partial\alpha}Prior_{\alpha} &= \left(\frac{1}{b} - 1\right)\cdot\frac{1}{\alpha}\\ \frac{\partial^2}{\partial\alpha^2}Prior_{\alpha} &= -\left(\frac{1}{b} - 1\right)\cdot\frac{1}{\alpha^2}\end{aligned} \tag{21}$$

Compute the derivatives of $Prior_{\alpha,b}$ - corresponding to h_{1b} Due to the form of the prior $Prior_{\alpha} = \log\left(\frac{1}{2b}\right) + \left(-\frac{1}{b} - 1\right)\log\alpha$ the only non-zero partial derivatives are:

$$\begin{aligned}\frac{\partial}{\partial\alpha}Prior_{\alpha} &= \left(-\frac{1}{b} - 1\right)\cdot\frac{1}{\alpha}\\ \frac{\partial^2}{\partial\alpha^2}Prior_{\alpha} &= -\left(-\frac{1}{b} - 1\right)\cdot\frac{1}{\alpha^2}\end{aligned} \tag{22}$$

As h_{1a} and h_{1b} differ only through the prior on the parameter α, the quantities above can be used to compute all h_{1a}, h_{1b}, and h_2 and their corresponding derivatives:

$$\begin{aligned}&h_{1a}(\alpha,\beta_0,\sigma^2) = -\frac{1}{n}(L_0(\alpha,\beta_0,\sigma^2) + L_1(\alpha,\beta_0,\sigma^2) + L_2(\alpha,\beta_0,\sigma^2) + L_3(\alpha,\beta_0,\sigma^2) + Prior_{\sigma^2} + Prior_{\alpha,a})\\ &h_{1b}(\alpha,\beta_0,\sigma^2) = -\frac{1}{n}(L_0(\alpha,\beta_0,\sigma^2) + L_1(\alpha,\beta_0,\sigma^2) + L_2(\alpha,\beta_0,\sigma^2) + L_3(\alpha,\beta_0,\sigma^2) + Prior_{\sigma^2} + Prior_{\alpha,b})\\ &h_2(\alpha=1,\beta_0,\sigma^2) = -\frac{1}{n}(L_0(\alpha=1,\beta_0,\sigma^2) + L_1(\alpha=1,\beta_0,\sigma^2) + L_2(\alpha=1,\beta_0,\sigma^2) + L_3(\alpha=1,\beta_0,\sigma^2) +\\ &Prior_{\sigma^2})\end{aligned} \tag{23}$$
